# Effects of Internet-Based Dementia Risk Reduction Education on Risk and Protective Factor Knowledge, Intentions, and Health Behaviors: Randomized Controlled Trial

**DOI:** 10.2196/79405

**Published:** 2026-03-10

**Authors:** Anthony J Levinson, Stephanie Ayers, Sandra Clark, Rebekah Woodburn, Amy Schneeberg, Dima Hadid, Nick Kates, Gillian Strudwick, Roland Grad, Alexandra Papaioannou, Maureen Dobbins, Henry Siu, Dante Duarte, Karen Saperson, Sharon Marr, Doug Oliver, Sarah Neil-Sztramko

**Affiliations:** 1 Faculty of Health Sciences, McMaster University, 1280 Main Street West, Hamilton, ON, L8S4L8, Canada, 1 9055259140 ext 26094; 2 Faculty of Health Sciences, Geras Centre for Aging Research, 88 Maplewood Avenue, Hamilton, ON, Canada; 3 Centre for Addiction and Mental Health, Toronto, Canada; 4 Department of Family Medicine, McGill University, Montreal, Canada; 5 Faculty of Health Sciences, National Collaborating Centre for Methods and Tools, Hamilton, Canada; 6 Department of Medicine, Unity Health Toronto, Toronto, Canada; 7 Faculty of Health Sciences, National Collaborating Centre for Methods and Tools, Hamilton, ON, Canada

**Keywords:** web-based intervention, internet, eHealth, dementia risk, dementia prevention, Alzheimer disease, education and training, clinical trial, public health, digital health interventions

## Abstract

**Background:**

Dementia prevention through the reduction of modifiable risk factors is gaining attention as a public health strategy. However, public knowledge of dementia risk and protective factors remains low. Web-based education offers a potential solution to raise awareness and promote risk-reduction behaviors.

**Objective:**

This randomized controlled trial evaluated the effectiveness of DementiaRisk.ca, an internet-based multimedia educational intervention, in increasing knowledge of dementia risk factors, intentions to engage in risk reduction behaviors, and changes in health behaviors.

**Methods:**

A 2-arm randomized controlled trial was conducted with 510 participants (265 in the intervention group and 245 in the control group). Participants were randomized to receive either the e-learning about dementia risk and promoting brain health, which included a multimedia lesson and microlearning emails, or a control intervention focused on mild cognitive impairment. Outcomes included knowledge of dementia risk factors, intentions to engage in risk reduction, and health behaviors, measured at baseline (T1), 4 weeks (T2), and 2 months postintervention (T3). Outcomes were analyzed using linear mixed effects models with fixed effects for group, time, and their interaction, and a random intercept for participants.

**Results:**

Of the 510 randomized participants, 405 (79.4%) completed all intervention components. Participants were predominantly female (n=309, 60.6%) and aged 55 years or older (n=284, 55.7%). Baseline mean dementia knowledge scores were 17.0 (SD 5.5) in the intervention group and 17.4 (SD 6.0) in the control group. At T2, scores increased to 25.8 (SD 4.5) and 23.6 (SD 5.1), respectively, yielding a between-group difference of 2.2 points (95% CI 1.2‐3.2; *P*<.001), which was sustained at T3. Both groups showed significant improvements in knowledge, intentions, and health behaviors over time, with larger knowledge gains in the intervention group and particularly among participants with lower educational attainment. Intentions to engage in dementia risk reduction improved in both groups at T2 (intervention: +1.0, 95% CI 0.2‐1.8; control: +1.4, 95% CI 0.5‐2.3), with no significant between-group difference. Self-reported physical activity increased from 31.7 (SD 25.0) to 38.6 (SD 27.5) in the intervention group and from 29.9 (SD 23.5) to 32.5 (SD 26.6) in the control group, with a between-group difference of 5.4 points at T2 (95% CI 0.3‐10.5; *P*=.04). No significant between-group differences were observed for diet, alcohol use, or other health behaviors. Qualitative findings indicated that participants valued the intervention for improving awareness of dementia risk factors, motivating proactive lifestyle changes, and enhancing confidence in applying prevention information.

**Conclusions:**

This internet-based dementia risk reduction e-learning program improved dementia-related knowledge and increased self-reported physical activity, particularly among participants with lower educational attainment. Effects on other health behaviors were limited. These findings support the use of well-designed e-learning as a scalable public health strategy to strengthen dementia risk reduction literacy and encourage selected healthy behaviors.

## Introduction

### Background

As our population ages, the prevalence of dementia is expected to continue to rise. Globally, dementia affects over 55 million people, with annual costs to health care, long-term care, and informal care partners exceeding US $1.3 trillion [1-4]. Without effective prevention strategies, the number of dementia cases is projected to triple by 2050, representing a significant global health and economic burden. In Canada, it is estimated that the number of people living with dementia will triple over the next 30 years [4]. However, increasing research highlights that there are many actions individuals can take to reduce their risk of dementia. According to the recent Lancet Commission report, up to 45% of dementias could be prevented through a variety of modifiable factors [4]. Similar estimates have been reported from the Canadian Longitudinal Study on Aging, for the impact of 12 risk factors on dementia cases, with physical inactivity, depression, and hypertension being particularly important in the Canadian context [5]. Other longitudinal studies have found that engaging in up to 4‐5 healthy lifestyle behaviors may reduce the risk of developing Alzheimer dementia by 37% to 60% [6,7]. The Landmark Study, which developed evidence-based data modeling to demonstrate the impacts of improving risk reduction efforts to delay onset across the population, suggests that a delay of developing dementia by as little as one year could result in nearly 500,000 fewer cases of dementia by 2050 [8]. Additionally, if risk reduction efforts can delay the onset of dementia by 10 years, over 4 million new cases of dementia could be avoided by 2050 [8]. Initiatives to help prevent dementia may also prove to be cost-effective at the societal level while simultaneously allowing individuals to help themselves and for care partners to support their loved ones [9].

### Gaps in Knowledge

Despite growing evidence about modifiable risk factors, public understanding of dementia prevention remains limited. A national public opinion survey conducted by EKOS Research Associates Inc for the Public Health Agency of Canada revealed that only 37% of Canadians recognized chronic conditions such as hypertension, heart disease, and diabetes as risk factors for dementia [10]. Awareness of other significant factors, such as hearing loss, was even lower, with only over 10% identifying it as a risk. Meanwhile, misconceptions, such as overestimating the role of toxic chemicals, for which there is limited evidence, persist [10].

### Educational Interventions

Educational interventions have the potential to improve knowledge of dementia risk and promote health behavior change [11,12]. While much of the existing literature has focused on training health care providers, a smaller number of studies have examined public education initiatives, which typically involve synchronous, one-on-one delivery by professionals. Most existing resources are predominantly text-based and often lack interactivity or adherence to principles of instructional design [13].

Web-based education offers a promising alternative due to its scalability, flexibility, and accessibility. Evidence from other health domains shows that e-learning tools that incorporate multimedia elements, such as audio narration, visuals, and interactive quizzes, are more engaging and improve long-term knowledge retention compared to static, text-only formats [14-16]. However, most of the web-based content about modifying dementia risk factors is text-based, such as static web pages or online PDF pamphlets or booklets, often requires a substantial time commitment, is frequently associated with a commercial entity or product for sale, or requires real-time participation [14,17,18]. Moreover, few, if any, evidence-based multimedia interventions on this topic have been rigorously studied using randomized controlled trials (RCTs), particularly those freely accessible to the public in multiple languages.

### Objectives

The objectives of this study were to: (1) evaluate whether exposure to an e-learning intervention (DementiaRisk.ca) changes knowledge of dementia risk factors, intention to engage in risk reduction activities, and health behaviors related to dementia risk reduction. The primary outcome was the change in these variables from baseline (T1) to 4 weeks postintervention (T2), with secondary analyses examining whether changes were sustained at 2 months postintervention (T3); and (2) explore qualitative aspects such as participants’ engagement and satisfaction with the intervention, as well as barriers and facilitators to use and dissemination of the information provided by the intervention, in adults without cognitive impairment.

## Methods

### Study Design

This study used a sequential explanatory mixed methods design, consisting of a 2-arm RCT, followed by a qualitative thematic content analysis to explore the findings from the RCT in greater depth. Full details of the methods have been published [19]. No changes were made from the published protocol. A mixed methods design was chosen to incorporate both quantitative and qualitative analysis, which allowed for a more comprehensive and nuanced understanding of the relationship between the intervention and its impacts.

### Participants

Participants were recruited from March 2023 to April 2023 using AskingCanadians, a paid panel service to allow for the recruitment of a representative and diverse sample of Canadians. Eligible participants were English-speaking adults with access to email and the internet, aged 18 years or older who had never been diagnosed with dementia. Participants were provided with the study information and eligibility criteria through an online link. After meeting eligibility criteria, participants were redirected to our online research study platform to complete informed consent and baseline measures. Informed consent was obtained digitally, with participants required to review an online information page before consenting to participate. We attempted to recruit participants across a range of ages, as risk factors may impact people throughout their life course, and many risk factors often begin to accumulate in midlife, many years before cognitive signs or symptoms emerge [4]. Ethical approval was provided by the Hamilton Integrated Research Ethics Board (project ID 14886).

### Study Procedures

Following completion of online informed consent, participants were randomized and directed to their assigned intervention or control group learning path. Participants were randomized in a 1:1 ratio via a computer-generated sequence through our research platform using a permuted block stratified randomization, with education and age as the stratification variables.

To the best of our ability, efforts were made to blind participants to the study hypothesis and allocation group by referring to the study in broad terms, such as “e-learning about cognitive impairment and promoting brain health” (rather than explicitly “dementia prevention,” for example). The principal investigator (AJL) and data analysts (DH and AS) remained blinded to group allocation throughout the data collection and analysis phases.

### Intervention

**Figure 1.**
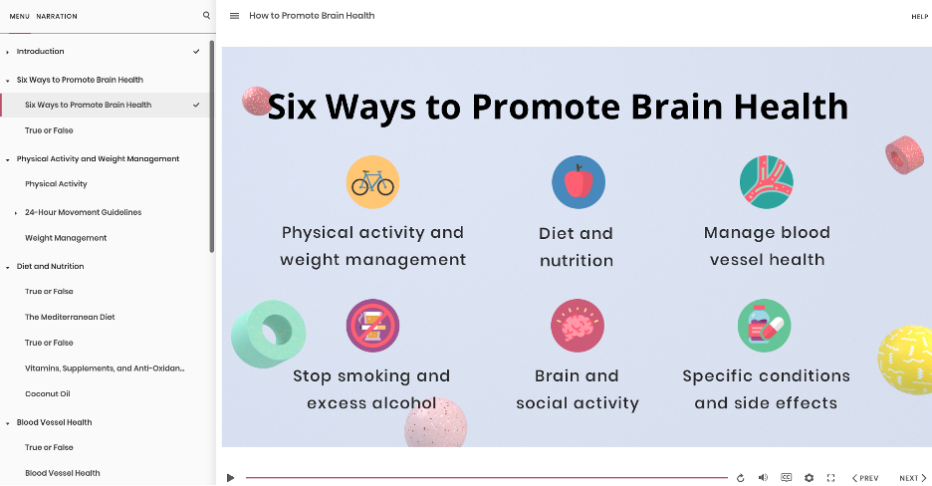
Screenshot of the DementiaRisk e-learning intervention.

**Figure 2.**
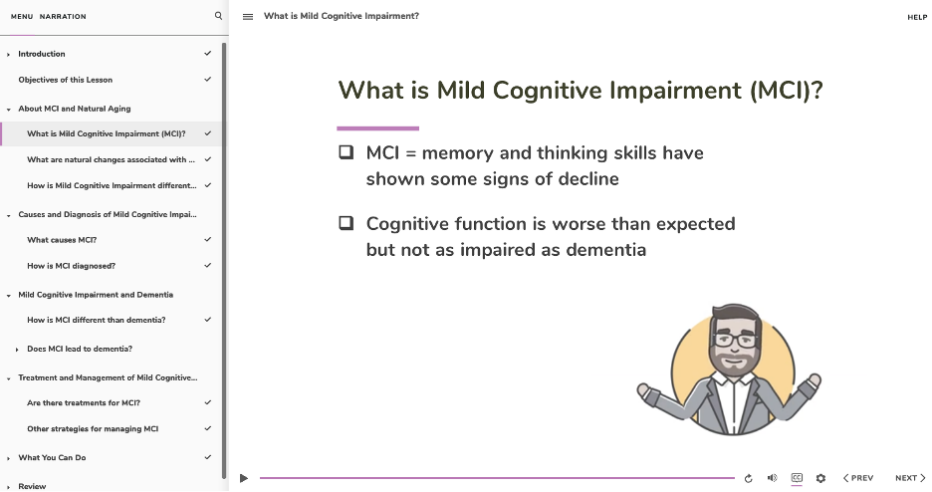
Screenshot from the control group e-learning.

The intervention group received e-learning about dementia risk reduction and promoting brain health consisting of the following components: (1) one 30-minute multimedia e-learning lesson (DementiaRisk.ca) about dementia risk and protective factors (Figure 1), and (2) a series of 12 prescheduled microlearning emails. The emails included small, focused segments of content taken directly from the e-learning lesson designed to reinforce key information from the e-learning lesson through spaced repetition.

Participants had 4 weeks to complete the e-learning lesson and received 3 emails per week during that period.

Content for the intervention was based predominantly on the World Health Organization (WHO) guidelines on risk reduction for cognitive decline and dementia and the Lancet Commission’s 2020 report on dementia prevention, intervention, and care [4,20]. Instructional design was guided by the Mayer Cognitive Theory of Multimedia Learning, ensuring that materials were delivered in manageable segments, using a combination of visuals, audio narration, and other evidence-based principles essential to effective e-learning that have been shown to enhance knowledge retention [13,14,16,17].

Other theoretical frameworks also informed the overall design of the intervention and its evaluation. The theory of planned behavior (TPB) [21,22] was used due to its extensive application with a number of dementia prevention risk factors (eg, smoking, diet, alcohol consumption, and physical activity) [23-27]. TPB suggests that intentions to engage in or change specific behaviors depend on motivation and ability across 6 constructs that collectively represent a person’s actual control over their behavior. DementiaRisk aimed to change participants’ attitudes and behavioral intentions by increasing knowledge related to dementia prevention and modifiable risk factors. The intervention and its evaluation were also informed by the Acquisition Cognition Application–Level of Outcomes model, which helps explain the value of the information from the user perspective, looking at the domains of situational relevance, cognitive impact, use of the information, and if used, any perceived health benefits [28].

### Control Condition

The control group participants received the following components: (1) 30-minute lesson on mild cognitive impairment (Figure 2), and (2) a series of 12 microlearning emails with small segments of content to reinforce material from the lesson. The design of the control group e-learning was also informed by similar principles and theoretical frameworks.

### Outcome Measures

Quantitative data were collected through online questionnaires at baseline (T1), 4 weeks (T2), and at 2 months postintervention (T3). Knowledge of dementia risk factors was collected using a 15-item multiple choice assessment aligned with the content of the intervention as well as the Risks and Health Promotion subdomain of the Dementia Knowledge Assessment Scale [29]. The total maximum score for this assessment is 30 (maximum 2 points per question), with higher scores indicating higher levels of knowledge related to dementia risk factors. Intentions to engage in health behaviors were measured using a 10-item assessment. The total maximum score for this assessment is 60, with a higher score indicating higher levels of intention to engage. Current participant health behaviors related to dementia risk factors were assessed using the Godin-Shephard Leisure-Time Physical Activity Questionnaire for physical activity [30] along with additional questions related to diet, smoking, alcohol consumption, social activity, traumatic brain injury, blood pressure, depression, air pollution, diabetes, sleep habits, brain activity, and hearing. Typical interpretation of total leisure activity scores uses a range of “insufficiently active or sedentary” (less than 14 points), “moderately active” (14‐23 points), and “active” (24 points or more).

Data related to engagement with the e-learning programs were collected during the active study phase to gain a clearer understanding of how the use of web-based learning throughout the study (or fidelity of delivery) might have impacted changes in knowledge, intentions, or health behaviors. The following metrics were used: number of e-learning starts and completions, e-learning completion rates, time spent on lessons, and microlearning email open rates.

All data were collected through secure password-protected online platforms, which automatically recorded responses and stored data. Participant responses were anonymized prior to analysis. Missing data were handled using linear mixed effects models, which incorporate all available data under the assumption of missing at random (MAR). Given the structure of the data and the analytic approach, multiple imputation was not performed, as there is evidence that linear mixed models (LMMs) provide unbiased estimates without imputation when using full information maximum likelihood estimation [31].

### Qualitative Data Collection

Qualitative data were collected from participants at T2 through both structured and open-ended questions using the Information Assessment Method for Patients and Consumers (IAM4all), a validated questionnaire that assesses outcomes of online consumer health information [19,32]. Qualitative questions explored participant satisfaction with the intervention, changes in attitudes, beliefs, and behaviors during the study, and feedback on proposed future dissemination methods. Data collection continued until no new themes emerged.

### Data Analysis

#### Quantitative Data

RStudio (version 4.4.1; R Foundation) was used for quantitative data analysis [33-36]. LMMs were used to examine the relationship between group, time point, and each of the 3 outcomes.

All models included outcome scores from all 3 time points: baseline (T1), 4 weeks (T2), and 2 months post-intervention (T3) as the dependent variable. An interaction between time, group, and participant ID was included as a random intercept. Random slopes were not included due to limited time points and sample size constraints. The point estimates and 95% CIs comparing groups at T2 were used to address the primary objective. Exploratory analysis of whether changes observed at T2 were sustained at T3 was also addressed through the inclusion of the group by time point interaction.

Sex, age group (<45, 45‐64, and ≥65 y), and education level (high school equivalent or less, some college or university, and college or university or graduate degree) were included as covariates in all models. Additionally, exploratory subgroup analyses of these were performed through testing interaction terms of group assignment and the stratification terms, as well as between time point and stratification terms. Nested models were compared using likelihood ratio tests (*P*<.05). For knowledge score only, having a family history of dementia or having been a care partner was combined into a single variable and tested as a potential effect modifier.

Missing data were explored across all 3 outcome variables: knowledge of dementia risk factors, intentions to engage in dementia risk reduction behaviors, and intentions to access dementia risk reduction information. For each outcome, participants with missing versus observed scores were compared at each timepoint across key demographic and exposure variables. Descriptive summaries and formal comparisons using chi-square or Fisher exact tests were conducted to assess potential differential attrition. Linear mixed effects models were used to incorporate all available data under the assumption of MAR, and multiple imputation was not performed.

All models were run for both intention-to-treat (ITT) and per-protocol (PP) group allotment. Effect sizes for group comparisons were calculated using the *emmeans* package in RStudio version 2023.12.1.402 [33-36]. Data analysts were blinded to participant grouping.

Using a conservative estimate of a small effect size (0.16, from a meta-analysis of internet health behavior change interventions), with a power of 0.80 and α of 0.05, we aimed to recruit a total of 388 participants in the study [37,38]. The sample size was increased by 25% to account for anticipated dropout, which brought the target recruitment number to 485 individuals [37,38]. These recruitment numbers and strategies have been used successfully in similar knowledge translation intervention studies and were a feasible target given the use of the paid panel [39].

#### Qualitative Data

For qualitative data, a conventional inductive content analysis approach was used [40]. All qualitative data were entered into NVivo (version 14; Lumivero) [41,42]. Data were systematically examined [19]. Codes and themes were developed based on the content, with no reliance on preconceived theories. The primary analyst (DH) conducted the initial coding and generated preliminary themes to ensure consistency and depth in the interpretation of the data. To strengthen rigor and minimize bias, the primary coder (DH) met weekly with the principal investigator (AJL) and other members of the research team to discuss developing codes and themes, confirm interpretations, and ensure analytic dependability. This approach has been shown to enhance analytic rigor through peer debriefing and triangulation [31]. The analysis focused on each theme separately. An iterative process of confirmation and reflection was used to analyze the data collaboratively. An audit trail was maintained, including memos from each stage of data collection and analysis. Findings of themes were triangulated between analysts. We ensured the study’s trustworthiness by considering validity standards that ensure rigor, confirmability, credibility, dependability, and transferability.

### Data Integration

Although the quantitative and qualitative components were analyzed separately, integration occurred during interpretation. The quantitative findings demonstrated changes in dementia risk–related knowledge, behavioral intentions, and selected health behaviors over time, while the qualitative findings helped contextualize these outcomes by elucidating participants’ perceptions of the intervention’s relevance, understandability, and anticipated benefits. In particular, qualitative data provided insight into how increased awareness translated into motivation for short-term lifestyle changes and highlighted perceived facilitators and barriers to applying dementia risk–reduction information. This sequential explanatory mixed methods approach enabled a more comprehensive understanding of the intervention’s impact than either quantitative or qualitative findings alone.

### Ethical Considerations

This study gained Hamilton Integrated Research Ethics Board approval on August 24, 2022 (project ID 14886). Participants completed all study components entirely online. Participants were required to provide informed consent and were informed of the length of time of the e-learning, surveys, and email campaign as well as informed about details surrounding data collection, storage, and investigator identities. Participants’ identities and confidentiality were maintained throughout the research study. All participant data were deidentified, and all findings will be nonidentifiable. There was no known risk or harm to participating in this study or publicizing its results or findings. Participants were provided with AskingCanadians points that could be redeemed for various gift cards as compensation for participation in the study.

## Results

### Participant Characteristics

**Figure 3.**
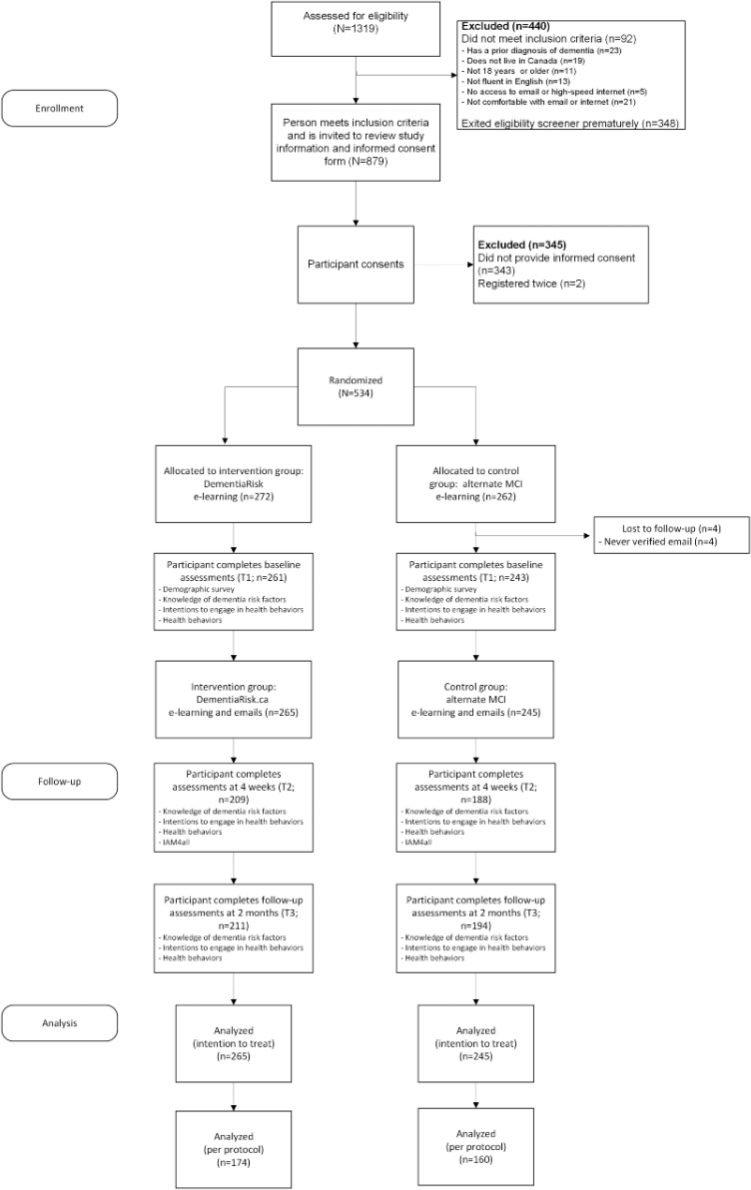
CONSORT (Consolidated Standards of Reporting Trials) study flow diagram. Note that participants were able to access all assessments at various time points, regardless of whether or not they completed previous components and were therefore not considered lost to follow-up. MCI: mild cognitive impairment.

A total of 1319 individuals responded to the initial online recruitment message and 879 eligible participants were invited to complete informed consent. 534 individuals consented and were randomly assigned to the intervention (n=272) or control group (n=262). A total of 510 participants began the study (intervention n=265; control n=245). Retention at T3 was 79.4% (intervention n=211; control n=194). Participant flow is detailed in Figure 3. Participants were able to access all assessments at various time points, regardless of whether they had completed previous components and were therefore not considered lost to follow-up (see Checklist 1).

Baseline characteristics were similar across all groups (all *P*>.05). Table 1 presents the demographic details of participants in each group. Participants were predominantly older (55.7%, aged ≥55 y), female (60.6%), and in good, very good, or excellent health (81.2%). The majority of participants identified as Caucasian (55.3%) followed by Chinese (10.4%) and South Asian or multiracial (each at 6.1%). Distribution patterns were relatively consistent across groups, though the control group had a slightly higher proportion of multiracial and Indigenous or First Nations participants. When asked about a family history of dementia, 31.4% of participants reported having a family history, 51.6% reported no family history, and 16.1% were unsure. The responses were consistent between groups. Finally, approximately one-quarter of participants (25.1%) identified as being a dementia care partner, with a higher proportion in the intervention group (27.9%) compared to the control group (22%).

**Table 1.** Participant demographics (N=510).

Characteristics	Intervention (n=265), n (%)	Control (n=245), n (%)	Overall (N=510), n (%)
Sex			
Male	107 (40.4)	89 (36.3)	196 (38.4)
Female	155 (58.5)	154 (62.9)	309 (60.6)
Missing	3 (1.1)	2 (0.8)	5 (1)
Age (y)			
18-24	1 (0.4)	3 (1.2)	4 (0.8)
25-34	26 (9.8)	23 (9.4)	49 (9.6)
35-44	41 (15.5)	37 (15.1)	78 (15.3)
45-54	51 (19.2)	44 (18)	95 (18.6)
55-64	53 (20)	58 (23.7)	111 (21.8)
65-74	59 (22.3)	60 (24.5)	119 (23.3)
≥75	31 (11.7)	18 (7.3)	49 (9.6)
Missing	3 (1.1)	2 (0.8)	5 (1)
Race and ethnicity			
Asian	7 (2.6)	10 (4.1)	17 (3.3)
Asian, East Asian	1 (0.4)	2 (0.8)	3 (0.6)
Black or African	8 (3)	6 (2.4)	14 (2.7)
Canadian	11 (4.2)	11 (4.5)	22 (4.3)
Caribbean	0 (0)	1 (0.4)	1 (0.2)
Chinese	28 (10.6)	25 (10.2)	53 (10.4)
East Asian	1 (0.4)	1 (0.4)	2 (0.4)
Eastern European	1 (0.4)	0 (0)	1 (0.2)
European	4 (1.5)	3 (1.2)	7 (1.4)
Filipino	1 (0.4)	2 (0.8)	3 (0.6)
Indigenous or First Nations	1 (0.4)	6 (2.4)	7 (1.4)
Jewish	3 (1.1)	3 (1.2)	6 (1.2)
Latino	2 (0.8)	0 (0)	2 (0.4)
Middle Eastern	1 (0.4)	0 (0)	1 (0.2)
Multiracial	11 (4.2)	20 (8.2)	31 (6.1)
South Asian	20 (7.5)	11 (4.5)	31 (6.1)
Western European	12 (4.5)	10 (4.1)	22 (4.3)
White or Caucasian	150 (56.6)	132 (53.9)	282 (55.3)
Missing	3 (1.1)	2 (0.8)	5 (1)
Health status			
Excellent	16 (6)	14 (5.7)	30 (5.9)
Very good	83 (31.3)	68 (27.8)	151 (29.6)
Good	124 (46.8)	109 (44.5)	233 (45.7)
Fair	38 (14.3)	48 (19.6)	86 (16.9)
Poor	1 (0.4)	4 (1.6)	5 (1)
Missing	3 (1.1)	2 (0.8)	5 (1)
Family history of dementia			
I am not sure	44 (16.6)	38 (15.5)	82 (16.1)
No	136 (51.3)	127 (51.8)	263 (51.6)
Yes	82 (30.9)	78 (31.8)	160 (31.4)
Missing	3 (1.1)	2 (0.8)	5 (1)
Dementia care partner			
No	188 (70.9)	189 (77.1)	377 (73.9)
Yes	74 (27.9)	54 (22)	128 (25.1)
Missing	3 (1.1)	2 (0.8)	5 (1)

### e-Learning Program Engagement

Metrics related to participant engagement with the e-learning programs are presented in Table 2. Overall, participants had a high e-learning lesson completion rate of 93% for both the intervention and control groups. Participants spent roughly the expected time on the e-learning lessons: an average of 31 minutes and 52 seconds (SD 17 minutes and 47 seconds) on the intervention lesson (estimated time to complete was 30‐35 min), and an average of 26 minutes and 11 seconds (SD 10 minutes 37 seconds) on the control lesson (estimated time to complete was 25‐30 min). The microlearning email campaign open rates were also strong (80.5% in the intervention and 78.6% in the control).

**Table 2.** e-Learning metrics.

e-Learning metric	Intervention (n=265)	Control (n=245)
Lesson starts	235	223
Lesson completions	218	207
Lesson completion rate (%)^a^	93	93
Average time spent on lesson	31 min, 52 s^b^	26 min 11 s
Micro-learning email open rate (%)	80.5	78.6

a Lesson completion rate is defined as lesson completions divided by lesson starts.

b Participants who spent over 1.5 hours on the module (n=20) who may have left their computer on after completing the lesson were removed from this average.

### Changes in Knowledge, Intentions, and Health Behaviors

The results of the linear mixed effects model revealed no significant difference between groups in baseline knowledge, intentions to engage in health behaviors, or health behaviors’ scores. The results of all models can be found in Multimedia Appendix 1.

### Knowledge of Dementia Risk Factors

**Figure 4.**
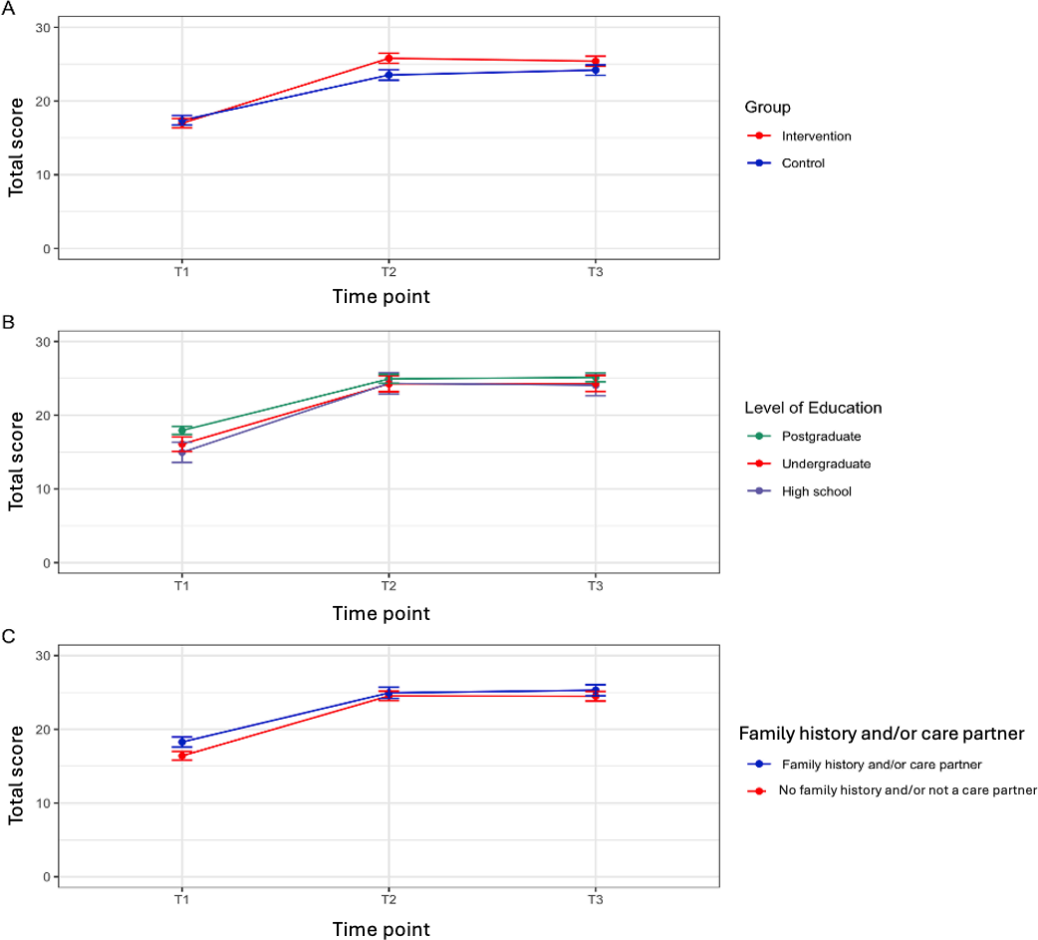
Marginal means from linear mixed model (LMM) with 95% CI for knowledge score by: (A) group, (B) level of education, and (C) dementia history or care partner.

Knowledge scores did not differ between groups at baseline (estimate −0.39, 95% CI −1.29 to 0.51; *P*=.40). Both groups improved significantly from T1 to T2, but the intervention group showed a statistically significantly greater increase. The difference at T2 was 2.66 points in favor of the intervention (95% CI 1.70‐3.63; *P*<.001). Knowledge gains were maintained at T3, with the intervention group continuing to exhibit significantly greater gain (estimate 1.60, 95% CI 0.65‐2.55; *P*=.001). Crude scores are shown in Table 3 and marginal means in Figure 4.

**Table 3.** Crude knowledge scores across time points by group (N=510).

	Intervention (n=265)			Control (n=245)			Overall (N=510)		
	T1	T2	T3	T1	T2	T3	T1	T2	T3
Mean (SD)	17.0 (5.51)	25.8 (4.54)	25.6 (4.68)	17.4 (5.98)	23.6 (5.13)	24.2 (5.06)	17.2 (5.74)	24.8 (4.95)	24.9 (4.91)
Median (IQR)	17.0 (14.0-20.0)	28.0 (24.0-29.0)	27.0 (23.0-29.0)	17.0 (13.0- 22.0)	25.0 (21.0- 28.0)	25.0 (21.0- 28.0)	17.0 (12.0- 21.0)	26.0 (22.0- 28.0)	26.0 (22.0- 29.0)
Missing, n (%)	3 (1.1)	56 (21.1)	54 (20.4)	3 (1.2)	57 (23.3)	51 (20.8)	6 (1.2)	113 (22.2)	105 (20.6)

Subgroup analysis indicated that participants with lower levels of education achieved the greatest gains at both T2 (estimate 2.36, 95% CI 0.83‐3.88; *P*=.003) and T3 (estimate 1.90, 95% CI 0.41‐3.39; *P*=.01). Those with a family history of dementia or caregiving experience began with higher baseline knowledge (estimate 1.88, 95% CI 0.96‐2.79; *P*<.001) and improved less over time, suggesting a ceiling effect. No significant interactions between group assignment and stratification variables were observed. Missing data analyses supported the assumption that data were missing at random.

### Intentions to Engage in Risk Reduction

**Figure 5.**
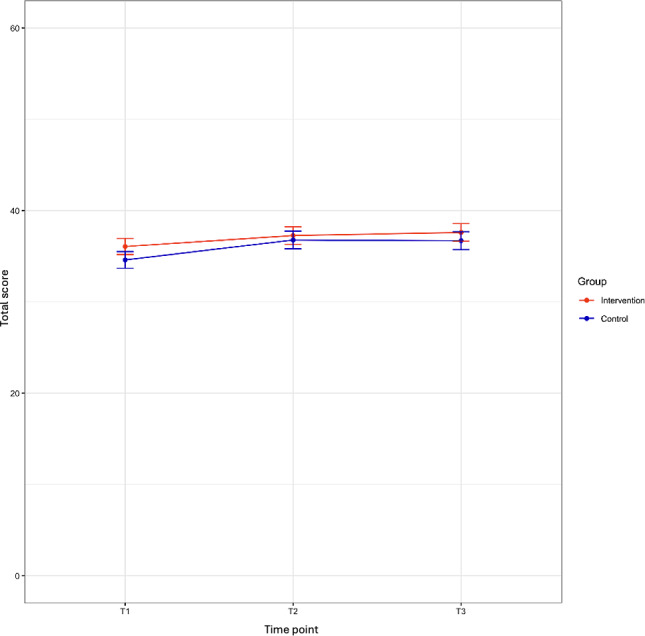
Intentions to engage in risk reduction score by level of education and group.

Intention scores differed at baseline, with higher scores in the intervention group. Both groups had a modest increase in their intentions from T1 to T2; the control group’s improvement was significant (estimate 1.42, 95% CI 0.18‐2.68; *P*=.03). However, there were no significant between-group differences at T2 (estimate −0.31, 95% CI −1.34 to 0.72; *P*=.55) or T3 (Multimedia Appendix 1; Figure 5). Table 4 provides crude intentions scores. Subgroup analyses did not identify significant interactions, and missingness followed an MAR pattern.

**Table 4.** Crude intentions scores across time points by group (N=510).

	Intervention (n=265)			Control (n=245)			Overall (N=510)		
	T1	T2	T3	T1	T2	T3	T1	T2	T3
Mean (SD)	37.5 (6.41)	39.2 (6.53)	39.3 (6.13)	36.2 (7.76)	37.6 (7.19)	38.1 (7.62)	36.9 (7.12)	38.4 (6.89)	38.7 (6.92)
Median (IQR)	38.0 (34.0- 42.0)	40.0 (35.0- 44.0)	40.0 (36.0- 43.0)	37.0 (31.5- 42.0)	39.0 (34.0- 43.0)	40.0 (34- 44.0)	38.0 (32.0- 42.0)	40.0 (34.0- 44.0)	40.0 (35.0- 44.0)
Missing, n (%)	35 (13.2)	77 (29.1)	78 (29.4)	26 (10.6)	74 (30.2)	66 (26.9)	61 (12)	151 (29.6)	144 (28.2)

### Health Behavior Changes: Godin-Shephard Leisure-Time Physical Activity Questionnaire

**Figure 6.**
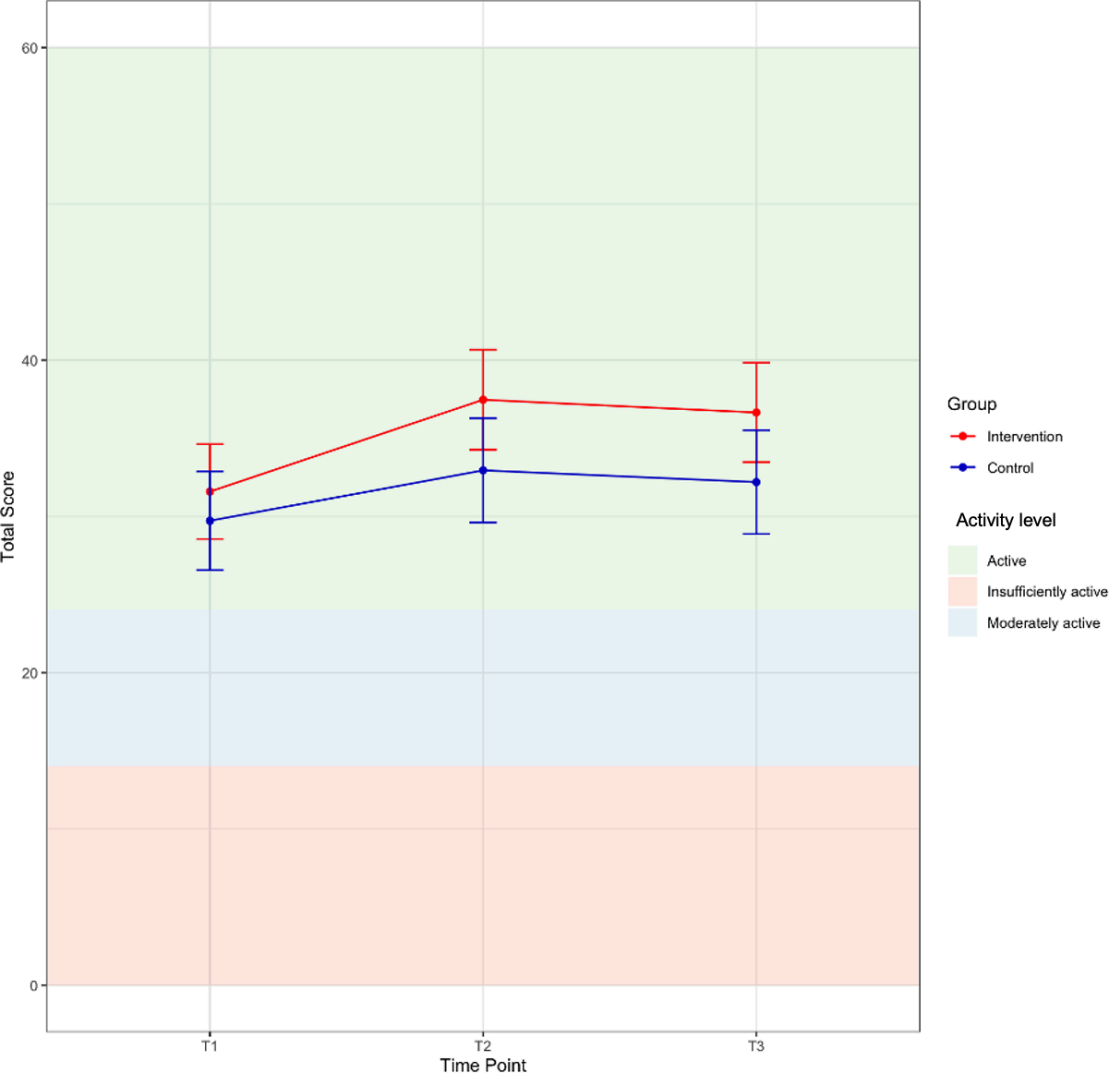
Linear mixed model (LMM) estimates and 95% CI for Godin-Shephard Leisure-Time Physical Activity Questionnaire score by group.

Self-reported physical activity levels did not differ between groups at baseline (estimate 1.87, 95% CI −2.52 to 6.25; *P*=.40). Both groups increased activity over time; the control group’s activity rose significantly at T2 (estimate 3.23, 95% CI 0.81‐5.64; *P*=.009) and was maintained at T3 (estimate 2.47, 95% CI 0.09‐4.86; *P*=.04). Although the intervention group showed numerically greater gains, the group× time interactions were not statistically significant at either T2 (estimate 2.65, 95% CI −0.69 to 5.99; *P*=.12) or T3 (estimate 2.59, 95% CI −0.72 to 5.89; *P*=.13). Crude values are shown in Table 5, with modeled estimates in Figure 6. Results of all models are shown in Multimedia Appendix 1.

**Table 5.** Crude Godin-Shephard Leisure-Time Physical Activity scores across time points by group (N=510).

	Intervention (n=265)			Control (n=245)			Overall (N=510)		
	T1	T2	T3	T1	T2	T3	T1	T2	T3
Mean (SD)	31.7 (25.0)	38.6 (27.5)	37.7 (27.1)	29.9 (23.5)	32.5 (26.6)	32.5 (26.5)	30.8 (24.3)	35.7 (27.2)	35.2 (26.9)
Median (IQR)	24.0 (14.0-46.8)	32.0 (19.0- 56.0)	33.5 (17.0- 50.8)	26.0 (14.0- 40.0)	27.0 (12.0- 44.2)	26.0 (15.0- 42.0)	24.5 (14.0- 44.0)	30.0 (15.0- 49.0)	28.0 (15.8- 48.0)
Missing, n (%)	3 (1.1)	56 (21.1)	55 (20.8)	3 (1.2)	57 (23.3)	51 (20.8)	6 (1.2)	113 (22.2)	106 (20.8)

Other health behavior measures, including diet, alcohol use, sleep, and social activity, showed no significant between-group differences (Multimedia Appendix 2). PP analyses aligned with the ITT results.

### IAM4all Outcomes

Using the IAM4all questionnaire at T2, participants in both groups rated the information as relevant or very relevant (intervention 94.1%, n=209; control 91.5%, n=194), easy to understand (intervention 100%, n=222; control 100%, n=212), and beneficial (intervention 95.9%, n=213; control 91%, n=193). Nearly all participants indicated that they intend to use what they have learned (intervention 98.2%, n=218; control 97.6%, n=207), and anticipate using the information to better understand dementia prevention (intervention 60.3%, n=134; control 81.6%, n=173), discuss with someone else (intervention 63.5%, n=141; control 55.6%, n=118), or do things differently (intervention 62.1%, n=138; control 35.3%, n=75). These quantitative findings help to contextualize the qualitative themes reported in the “Qualitative Findings” section.

### Qualitative Findings

Qualitative analysis was conducted inductively to generate themes and subthemes. A full list of qualitative themes and subthemes is provided in Multimedia Appendix 3. Overall, qualitative responses converged with data from the IAM4all, indicating that participants perceived the content as relevant, understandable, and actionable, with anticipated benefits for brain health and dementia risk reduction.

#### Relevance and Understandability

Participants commonly described the information as personally meaningful and focused on gaining a clearer understanding of dementia, including symptoms, early signs, and modifiable risk factors. Many emphasized the value of being able to recognize dementia symptoms and respond appropriately.

*Recognize symptoms of dementia and learn the best ways to cope*

[ID 551]

Participants also demonstrated a strong interest in recognizing the early signs of dementia to facilitate timely intervention. They displayed significant concern about the condition’s risk factors and root causes, aiming to make informed lifestyle choices to mitigate or delay its onset.

*Learn how to reduce the risk of dementia, strategies to delay the onset and minimize the symptoms of, dementia. Be able to recognize the signs of the onset of dementia.*

[ID 298]

#### Intended Use and Action

Responses frequently demonstrated a proactive stance towards health, particularly in adopting lifestyle changes that could potentially delay or prevent the onset of dementia.

*I will try to implement changes in my life based on what I learned in this.*

[ID 248]

They were highly motivated to integrate new knowledge into their daily routines, making informed decisions related to diet, exercise, and mental health practices.

*Motivates me to try and do more healthy things.*

[ID 163]

*Get motivated to do more walking, with the dogs. Maybe playing more cards with friends. I already play darts 2x a week and keep score in all games; adding and subtracting in my head while standing by the boards. Possibly learn how to play Sudoku, instead of just doing the crossword puzzles.*

[ID 332]

Participants demonstrated a strong commitment to applying what they learned, with many initiating medical check-ups, adjusting their diets, and incorporating regular physical activity as direct responses to the information provided.

*As a result of the information about the high correlation between hearing loss and dementia (higher than many other risk factors), I have made an appointment for ear wax removal, and will speak to the professional about hearing loss. Everyone should read the excellent information provided in the email campaign and surveys.*

[ID 68]

*Will go back on healthy diet, reduce sugar intake, get more exercise, more social activity.*

[ID 328]

Participants frequently described increased awareness, motivation, and intentions to make lifestyle changes, and some reported early self-initiated actions.

*This module did teach me somewhat more of what I need to do to keep myself and control my mental health, as well as physical health as I age. At 68, I do admit that I need to follow a better diet to help control my diabetes. As Emergency Medical Technicians, we were taught that we treated all causes of Dementia the same, as we could not differentiate between Diabetic, Alzheimer’s or anything else...they were all forms of some mental health. Having seen the decline of my former Spouse into the Diabetic Dementia, and his refusal to even try to modify any of his routine: I know that I must make some changes to my lifestyle.*

[ID 332]

#### Perceived Benefit

Beyond prevention, some participants valued learning about broader health connections, as well as current and emerging treatments. They appreciated the educational value of the study, which enhanced their understanding of brain health and its connections to other health issues to support more informed health and caregiving decisions.

*I would never have believed that hearing loss might be a sign of dementia.*

[ID 446]

#### Suggestions for Improvement

Participants also identified a desire for more comprehensive content, including practical steps for prevention and management, indicating areas for improving user experience and engagement in future studies.

*I think that some of the suggestions for potential action could use more direction as to sources to engage in action - websites. Other information to enable people to follow up actively.*

[ID 51]

The analysis further highlighted sex-specific interests, with female participants showing interest in caregiving strategies while male participants focused more on genetic risks. Additionally, across different age groups, there was a shift from a general focus on prevention in younger participants to practical management and caregiving in older adults, with a consistent appreciation for the informative impact of the study across all demographics.

## Discussion

### Principal Findings

The findings from this RCT demonstrate the effectiveness of the DementiaRisk internet-based education program in enhancing participants’ knowledge of dementia risk and protective factors. These results were statistically significant at both the 4-week and 2-month postintervention follow-up assessments, reflecting sustained improvements post-intervention.

While both groups improved, the intervention group demonstrated significantly greater gains. This highlights the value of health education in general, but underscores the importance of providing well-designed dementia risk reduction-specific content.

The intervention improved knowledge particularly among participants with lower education levels, suggesting that e-learning may help bridge knowledge gaps among populations with potentially less prior exposure to dementia-related information. Participants who identified as having a family history of dementia or as a care partner had higher levels of baseline knowledge of dementia. Gains among those without such experience indicate that the intervention may help inform individuals who have not been previously motivated by personal exposure to dementia, thereby expanding its potential reach and impact on the general population.

### Age Differences in Engagement With Preventative Measures

Older adults in our sample demonstrated greater intentions to engage in risk reduction behaviors compared to younger participants, which is consistent with prior evidence that perceived risk may increase motivation to adopt protective behaviors [43,44]. In contrast, younger adults may have lower perceived susceptibility to dementia, which can reduce their motivation to adopt lifestyle changes early in life [43,44].

Public health education efforts face unique challenges in engaging younger adults in reducing their risk of dementia. The concept of dementia as a ‘disease of aging’ contributes to a lack of urgency among younger populations [45]. Additionally, dementia prevention requires long-term behavior changes that may not provide immediate, tangible benefits, making it difficult to incentivize engagement in risk-reduction behaviors. Strategies such as emphasizing the broader benefits of lifestyle modifications (eg, improved cardiovascular health, mental well-being, and cognitive function) may help increase engagement among younger populations [44-47]. Framing dementia prevention within a broader “brain health” context, as suggested by the TPB, may also improve receptivity among younger individuals [48].

### Interpreting Intentions to Engage in Risk Reduction

The increase in intentions observed across both groups indicates that exposure to health-related e-learning, even when not specific to dementia-risk reduction, may positively influence motivation to adopt healthier behaviors. However, measuring health behavior intentions remains a challenge due to the lack of a widely accepted, validated tool specifically designed for assessing intentions related to dementia risk reduction. The TPB suggests that intentions are strong predictors of behavior, but this relationship can be influenced by external factors such as perceived barriers or social norms [22,49,50]. Given the absence of a standardized instrument for measuring intentions in this context, our study relied on a custom-developed measure. This introduces potential limitations in comparability with other studies. Furthermore, it is important to acknowledge the “intention-behaviour gap,” where individuals express a willingness to change but do not always follow through with action [50,51].

### Health Behavior Change and Practical Implications

An interesting finding was the increase in self-reported physical activity levels among the participants, which suggests that the educational content may have prompted short-term behavior change. The sustained increase in physical activity observed at both follow-up points (4 wk and 2 mo postintervention) aligns with previous research suggesting that e-learning platforms, when well-designed, can encourage behavior changes beyond mere knowledge acquisition [52]. Given that physical activity is a key modifiable risk factor for dementia, this result has promising implications for the broader public health goal of dementia prevention. While the increase in physical activity levels among intervention participants is noteworthy, it should be interpreted within the context of a short-term study. Many health behaviors, particularly those related to physical activity, require sustained effort over time to produce meaningful long-term changes [53]. Some risk factors such as traumatic brain injury or hearing loss are not easily modifiable within the time frame of a 3-month study or may require additional resources or support from health care providers [54]. This distinction highlights the importance of differentiating between modifiable and nonmodifiable risk factors when designing and interpreting public health interventions.

Our study’s findings align with existing research suggesting that short-term interventions are most effective for behaviors that require relatively low effort to change, such as increasing physical activity or making dietary improvements [55]. However, for more complex or chronic risk factors (eg, hypertension management and smoking cessation), longer-term interventions with ongoing reinforcement may be necessary to sustain behavior change.

Together, these results highlight that even brief, web-based interventions can produce measurable improvements in knowledge and short-term behavior, particularly when designed using evidence-based principles of instructional design. However, the practical significance and broader population impact of these changes warrant careful interpretation.

### Interpretation of Findings

Across outcomes, the impact and practical relevance of observed changes varied. Knowledge of dementia risk factors increased by approximately 30% from baseline. Although modest, this gain likely reflects a meaningful increase in dementia literacy—particularly because small, scalable improvements in public awareness can produce significant population-level benefits when applied broadly. While knowledge gains do not guarantee sustained behavior change, participants’ qualitative comments indicate that many viewed the information as personally important and, in some cases, actionable in the short term, supporting the practical relevance of the knowledge gain as a precursor to informed risk-reduction decisions.

Behavioral intentions to engage in dementia risk-reduction activities also increased throughout the study, but the absolute change was small (approximately 2%‐3%) and occurred in both groups. The qualitative findings help explain these results by demonstrating that participants primarily described changes in awareness, motivation, and short-term action, particularly for behaviors perceived as immediately feasible, such as getting a hearing test, walking, or dietary adjustments, while expressing uncertainty about how to implement more complex or long-term risk-reduction strategies. This pattern suggests that exposure to health-related online education may positively influence motivation to adopt healthier behaviors, though the clinical importance of this change remains uncertain. The use of a novel intentions measure further limits comparability across studies, highlighting the need for validated scales tailored to dementia prevention contexts. In contrast, self-reported leisure-time physical activity increased by roughly 20% among participants, which may represent a meaningful short-term gain, even if participants largely remained within the same physical activity category. These effects may have been smaller in magnitude because participants were already relatively active and health conscious at baseline.

### Instructional Design

One factor that may have contributed to the intervention’s effectiveness is the use of evidence-based principles of instructional design. These principles, based on the Mayer Cognitive Theory of Multimedia Learning, have been shown to positively impact learning outcomes and retention [14,17]. Applying multimedia design principles such as segmenting, personalization, self-testing with feedback, and worked examples may strengthen comprehension and engagement in e-learning [15,56]. While the majority of research has examined these principles in health professionals, our findings extend this work to other general public populations.

The micro-learning email campaign also incorporated the principles of spaced education and repetition. These principles have also been shown to be effective in enhancing knowledge retention in health care professionals, but do not appear to have been systematically studied with respect to health education for the public [57,58].

### Limitations and Future Directions

This study has several limitations. Although the sample was recruited from a representative panel of the Canadian population, it may not reflect populations with lower digital access or literacy. This could have introduced an element of selection bias, leading to higher baseline knowledge levels than anticipated. The sample also appeared relatively health-conscious, which may have limited the extent of behavior change. Additionally, all data were self-reported, which could introduce bias, especially in terms of behavior changes like physical activity. Finally, although a single coder performed the primary qualitative analysis, ongoing team-based verification and peer debriefing reduced potential bias and supported analytic consistency.

### Conclusions

The findings from this randomized controlled trial indicate that a multimedia, instructionally designed, internet-based dementia-risk program can significantly improve dementia-related knowledge, particularly among participants with lower levels of education. We also observed modest, short-term increases in self-reported intention to engage in dementia risk–reducing behaviors and in physical activity levels in the intervention group, although effects on other behaviors were limited. Given its scalability and accessibility, this type of e-learning intervention represents a promising public health strategy to strengthen dementia risk-reduction literacy and support selected healthy behaviors at scale. Future research should broaden the program’s reach and evaluate the durability of effects on knowledge, intentions, and health behaviors over time.

## Supplementary material

10.2196/79405 Multimedia Appendix 1 Results of all models.

10.2196/79405 Multimedia Appendix 2 Health behavior data.

10.2196/79405 Multimedia Appendix 3 Qualitative themes and subthemes.

10.2196/79405 Checklist 1 CONSORT-EHEALTH checklist.

## Abbreviations

CONSORT: Consolidated Standards of Reporting Trials
CONSORT-eHEALTH: Consolidated Standards of Reporting Trials of Electronic and Mobile Health Applications and Online Telehealth
IAM4all: Information Assessment Method for Patients and Consumers
ITT: intention-to-treat
LMM: linear mixed model
MAR: missing at random
PP: per protocol
RCT: randomized controlled trial
TPB: theory of planned behavior
WHO: World Health Organization
