# Supplementary material for: Effects of Internet-Based Dementia Risk Reduction Education on Risk and Protective Factor Knowledge, Intentions, and Health Behaviors: Randomized Controlled Trial
Source: J Med Internet Res. 2026 Mar 10;28:e79405. doi: 10.2196/79405 (PMC12975120; doi:10.2196/79405)
Supplement: Multimedia Appendix 3 [file jmir-v28-e79405-s003.docx]

| Themes | Subthemes |
| --- | --- |
| Positive feedback on study impact and information provided |  |
|  | Appreciation for knowledge gained |
|  | Encouraged participants to take proactive steps |
|  | Encouraged self-check up with specialist |
|  | Positive technical feedback |
| Requests for additional topics |  |
|  | Account for additional health factors |
|  | Add individual sex habit |
|  | Coping strategies |
|  | Explanation of the causes of dementia |
|  | Impact of external factors |
|  | Include practical steps for injury prevention |
|  | Interest in understanding the broader objectives and potential impacts |
|  | Lack of questions about current level of cognitive functioning |
|  | Lack of questions about eyesight |
|  | More direct advice or resources on managing specific health concerns |
|  | More information on prevention |
| Suggestions for improvement |  |
|  | Nothing to change |
|  | Availability of handouts |
|  | Broader dissemination of information |
|  | Clear definition or explanation of key terms |
|  | Clear wording of questions |
|  | Clearer communication of survey objectives |
|  | Feasibility |
|  | Long-term follow-up |
|  | More interactive learning experiences and validation of understanding |
|  | Request for additional resources/topics |
|  | Need for more specificity in questions related to family health history |
|  | No quizzes |
|  | Proactive approach for deeper engagement |
| Technical suggestions |  |
|  | Additional context to answers |
|  | Confusion caused by emails |
|  | Pacing and format |
|  | Improve designing study material (more inclusive) |
|  | More reminders of important deadlines and dates |
|  | More time to complete survey |
|  | Reduce time between surveys |
| Tracking health related activities and metrics |  |
| Use of cautious language |  |
| Information participants are hoping to learn from the online training |  |
|  | Impact of family history |
|  | General understanding and awareness of dementia |
|  | Debunk misinformation |
|  | Skills to manage dementia |
|  | Support strategies for caregivers |
| Motivation to learn |  |
|  | Interest in pursuing career in mental health or related field |
|  | Personal growth & knowledge |
|  | Relate to others |
| Personal concern-age related |  |
| Prevention and health promotion |  |
|  | Cognitive (brain) health |
|  | Preventive measures |
|  | Self-care and Informed lifestyle choices |
| Treatment and medical advances |  |
|  | Advanced knowledge and research |
|  | Impact of research and dissemination of knowledge |
|  | Treatment options |
| Understanding and identifying dementia |  |
|  | Causes |
|  | Early signs and symptoms |
|  | Emotional preparedness |
|  | Risk factors |
| How to use information |  |
|  | Healthcare and medication |
|  | Increased knowledge and awareness |
| Personal health practices |  |
|  | Improve lifestyle |
|  | Self-check up |
