# Supplementary material for: Effects of Internet-Based Dementia Risk Reduction Education on Risk and Protective Factor Knowledge, Intentions, and Health Behaviors: Randomized Controlled Trial
Source: J Med Internet Res. 2026 Mar 10;28:e79405. doi: 10.2196/79405 (PMC12975120; doi:10.2196/79405)
Supplement: Checklist 1 [file jmir-v28-e79405-s004.pdf]

CONSORT-EHEALTH (V 1.6.1) - Submission/Publication Form

The CONSORT-EHEALTH checklist is intended for authors of randomized trials evaluating web-based and Internet-based applications/interventions, including mobile interventions, electronic games (incl multiplayer games), social media, certain telehealth applications, and other interactive and/or networked electronic applications. Some of the items (e.g. all subitems under item 5 - description of the intervention) may also be applicable for other study designs.

The goal of the CONSORT EHEALTH checklist and guideline is to be
a) a guide for reporting for authors of RCTs,
b) to form a basis for appraisal of an ehealth trial (in terms of validity)

CONSORT-EHEALTH items/subitems are MANDATORY reporting items for studies published in the Journal of Medical Internet Research and other journals / scientific societies endorsing the checklist.

Items numbered 1., 2., 3., 4a., 4b etc are original CONSORT or CONSORT-NPT (non-pharmacologic treatment) items.
Items with Roman numerals (i., ii, iii, iv etc.) are CONSORT-EHEALTH extensions/clarifications.

As the CONSORT-EHEALTH checklist is still considered in a formative stage, we would ask that you also RATE ON A SCALE OF 1-5 how important/useful you feel each item is FOR THE PURPOSE OF THE CHECKLIST and reporting guideline (optional).

Mandatory reporting items are marked with a red *.
In the textboxes, either copy & paste the relevant sections from your manuscript into this form - please include any quotes from your manuscript in QUOTATION MARKS,
or answer directly by providing additional information not in the manuscript, or elaborating on why the item was not relevant for this study.

YOUR ANSWERS WILL BE PUBLISHED AS A SUPPLEMENTARY FILE TO YOUR PUBLICATION IN JMIR AND ARE CONSIDERED PART OF YOUR PUBLICATION (IF ACCEPTED).
Please fill in these questions diligently. Information will not be copyedited, so please use proper spelling and grammar, use correct capitalization, and avoid abbreviations.

DO NOT FORGET TO SAVE AS PDF _AND_ CLICK THE SUBMIT BUTTON SO YOUR ANSWERS ARE IN OUR DATABASE !!!

Citation Suggestion (if you append the pdf as Appendix we suggest to cite this paper in the caption):
Eysenbach G, CONSORT-EHEALTH Group

CONSORT-EHEALTH: Improving and Standardizing Evaluation Reports of Web-based and Mobile Health Interventions
J Med Internet Res 2011;13(4):e126
URL: http://www.jmir.org/2011/4/e126/
doi: 10.2196/jmir.1923
PMID: 22209829

stephanie.ayers@machealth.ca Switch account

Not shared

Draft saved

* Indicates required question

Your name *

First Last

Stephanie Ayers

Primary Affiliation (short), City, Country *

University of Toronto, Toronto, Canada

McMaster University, Hamilton, Canada

Your e-mail address *

abc@gmail.com

ayerss@mcmaster.ca

Title of your manuscript *

Provide the (draft) title of your manuscript.

Effects of Internet-Based Dementia Risk Reduction Education on Risk and Protective Factor Knowledge, Intentions, and Health Behaviours: A Randomized Controlled Trial

Name of your App/Software/Intervention *

If there is a short and a long/alternate name, write the short name first and add the long name in brackets.

DementiaRisk (DementiaRisk.ca)

Evaluated Version (if any)

e.g. "V1", "Release 2017-03-01", "Version 2.0.27913"

February 19, 2023

Language(s) *

What language is the intervention/app in? If multiple languages are available, separate by comma (e.g. "English, French")

English, French (RCT was conducted in EN onl[illegible]

URL of your Intervention Website or App

e.g. a direct link to the mobile app on app in appstore (itunes, Google Play), or URL of the website. If the intervention is a DVD or hardware, you can also link to an Amazon page.

https://dementiarisk.ca/

URL of an image/screenshot (optional)

https://dementiarisk.ca/

Accessibility *

Can an enduser access the intervention presently?

- (selected) access is free and open
- access only for special usergroups, not open
- access is open to everyone, but requires payment/subscription/in-app purchases
- app/intervention no longer accessible
- Other:

Primary Medical Indication/Disease/Condition *

e.g. "Stress", "Diabetes", or define the target group in brackets after the condition, e.g. "Autism (Parents of children with)", "Alzheimers (Informal Caregivers of)"

Dementia prevention

Primary Outcomes measured in trial *

comma-separated list of primary outcomes reported in the trial

Increase in knowledge of dementia risk factors

Secondary/other outcomes

Are there any other outcomes the intervention is expected to affect?

Intentions to engage in health behaviors, health behaviors related to behavior change for modifying dementia risk factors, e-learning analytics (progress or completion, login activity, and time on the lesson), email campaign analytics (open rate and click-through rate)

Recommended "Dose" *

What do the instructions for users say on how often the app should be used?

- ◯ Approximately Daily
- ◯ Approximately Weekly
- ◯ Approximately Monthly
- ◯ Approximately Yearly
- ◯ "as needed"
- ◉ Other: One-time e-learning module with 12-part email micro-learning series. I

Approx. Percentage of Users (starters) still using the app as recommended after 3 months *

- ◯ unknown / not evaluated
- ◯ 0-10%
- ◯ 11-20%
- ◯ 21-30%
- ◯ 31-40%
- ◯ 41-50%
- ◯ 51-60%
- ◯ 61-70%
- ◯ 71%-80%
- ◯ 81-90%
- ◯ 91-100%
- ◉ Other: Unknown how many RCT participants using as after 3-months; >300,0

Overall, was the app/intervention effective? *

- ◯ yes: all primary outcomes were significantly better in intervention group vs control
- ◯ partly: SOME primary outcomes were significantly better in intervention group vs control
- ◯ no statistically significant difference between control and intervention
- ◯ potentially harmful: control was significantly better than intervention in one or more outcomes
- ◯ inconclusive: more research is needed
- ◉ Other: Both groups showed significant improvements in knowledge, intentior

Article Preparation Status/Stage *

At which stage in your article preparation are you currently (at the time you fill in this form)

- ◯ not submitted yet - in early draft status
- ◯ not submitted yet - in late draft status, just before submission
- ◉ submitted to a journal but not reviewed yet
- ◯ submitted to a journal and after receiving initial reviewer comments
- ◯ submitted to a journal and accepted, but not published yet
- ◯ published
- ◯ Other:

Journal *

If you already know where you will submit this paper (or if it is already submitted), please provide the journal name (if it is not JMIR, provide the journal name under "other")

- ( ) not submitted yet / unclear where I will submit this
- (x) Journal of Medical Internet Research (JMIR)
- ( ) JMIR mHealth and UHealth
- ( ) JMIR Serious Games
- ( ) JMIR Mental Health
- ( ) JMIR Public Health
- ( ) JMIR Formative Research
- ( ) Other JMIR sister journal
- ( ) Other:

Is this a full powered effectiveness trial or a pilot/feasibility trial? *

- ( ) Pilot/feasibility
- (x) Fully powered

Manuscript tracking number *

If this is a JMIR submission, please provide the manuscript tracking number under "other" (The ms tracking number can be found in the submission acknowledgement email, or when you login as author in JMIR. If the paper is already published in JMIR, then the ms tracking number is the four-digit number at the end of the DOI, to be found at the bottom of each published article in JMIR)

- ( ) no ms number (yet) / not (yet) submitted to / published in JMIR
- (x) Other: 79405

TITLE AND ABSTRACT

1a) TITLE: Identification as a randomized trial in the title

1a) Does your paper address CONSORT item 1a? *

I.e does the title contain the phrase "Randomized Controlled Trial"? (if not, explain the reason under "other")

- (selected) yes
- Other:

1a-i) Identify the mode of delivery in the title

Identify the mode of delivery. Preferably use “web-based” and/or “mobile” and/or “electronic game” in the title. Avoid ambiguous terms like “online”, “virtual”, “interactive”. Use “Internet-based” only if Intervention includes non-web-based Internet components (e.g. email), use “computer-based” or “electronic” only if offline products are used. Use “virtual” only in the context of “virtual reality” (3-D worlds). Use “online” only in the context of “online support groups”. Complement or substitute product names with broader terms for the class of products (such as “mobile” or “smart phone” instead of “iphone”), especially if the application runs on different platforms.

	1	2	3	4	5	
subitem not at all important	○	○	○	○	○	essential

Does your paper address subitem 1a-i? *

Copy and paste relevant sections from manuscript title (include quotes in quotation marks "like this" to indicate direct quotes from your manuscript), or elaborate on this item by providing additional information not in the ms, or briefly explain why the item is not applicable/relevant for your study

"Effects of Internet-Based Dementia Risk Reduction Education"

1a-ii) Non-web-based components or important co-interventions in title

Mention non-web-based components or important co-interventions in title, if any (e.g., “with telephone support”).

	1	2	3	4	5	
subitem not at all important	○	○	○	○	○	essential

Does your paper address subitem 1a-ii?

Copy and paste relevant sections from manuscript title (include quotes in quotation marks "like this" to indicate direct quotes from your manuscript), or elaborate on this item by providing additional information not in the ms, or briefly explain why the item is not applicable/relevant for your study

"Effects of Internet-Based Dementia Risk Reduction Education"; we feel it is not essential to mention other non-web-based components since "Internet-Based Intervention" captures both the online e-learning module and email campaign, from subitem 1a-i

1a-iii) Primary condition or target group in the title

Mention primary condition or target group in the title, if any (e.g., “for children with Type I Diabetes”) Example: A Web-based and Mobile Intervention with Telephone Support for Children with Type I Diabetes: Randomized Controlled Trial

	1	2	3	4	5	
subitem not at all important	○	○	○	○	○	essential

Does your paper address subitem 1a-iii? *

Copy and paste relevant sections from manuscript title (include quotes in quotation marks "like this" to indicate direct quotes from your manuscript), or elaborate on this item by providing additional information not in the ms, or briefly explain why the item is not applicable/relevant for your study

As this internet-based intervention is intended for adults in the general population, we feel it is not essential to state the specific population in the title

1b) ABSTRACT: Structured summary of trial design, methods, results, and conclusions

NPT extension: Description of experimental treatment, comparator, care providers, centers, and blinding status.

1b-i) Key features/functionalities/components of the intervention and comparator in the METHODS section of the ABSTRACT

Mention key features/functionalities/components of the intervention and comparator in the abstract. If possible, also mention theories and principles used for designing the site. Keep in mind the needs of systematic reviewers and indexers by including important synonyms. (Note: Only report in the abstract what the main paper is reporting. If this information is missing from the main body of text, consider adding it)

	1	2	3	4	5	
subitem not at all important	○	○	○	○	○	essential

Does your paper address subitem 1b-i? *

Copy and paste relevant sections from the manuscript abstract (include quotes in quotation marks "like this" to indicate direct quotes from your manuscript), or elaborate on this item by providing additional information not in the ms, or briefly explain why the item is not applicable/relevant for your study

"A two-arm RCT was conducted with 510 participants (265 in the intervention group and 245 in the control group). Participants were randomized to receive either the e-learning about dementia risk and promoting brain health, which included a multimedia lesson and micro-learning emails, or a control intervention focused on mild cognitive impairment."

1b-ii) Level of human involvement in the METHODS section of the ABSTRACT

Clarify the level of human involvement in the abstract, e.g., use phrases like “fully automated” vs. “therapist/nurse/care provider/physician-assisted” (mention number and expertise of providers involved, if any). (Note: Only report in the abstract what the main paper is reporting. If this information is missing from the main body of text, consider adding it)

	1	2	3	4	5	
subitem not at all important	○	○	○	○	○	essential

Does your paper address subitem 1b-ii?

Copy and paste relevant sections from the manuscript abstract (include quotes in quotation marks "like this" to indicate direct quotes from your manuscript), or elaborate on this item by providing additional information not in the ms, or briefly explain why the item is not applicable/relevant for your study

"An internet-based dementia prevention e-learning program effectively enhanced knowledge and promoted healthy behaviours related to dementia risk reduction. "

"Self-reported health behaviours, particularly physical activity, increased significantly among the intervention group.'

1b-iii) Open vs. closed, web-based (self-assessment) vs. face-to-face assessments in the METHODS section of the ABSTRACT

Mention how participants were recruited (online vs. offline), e.g., from an open access website or from a clinic or a closed online user group (closed usergroup trial), and clarify if this was a purely web-based trial, or there were face-to-face components (as part of the intervention or for assessment). Clearly say if outcomes were self-assessed through questionnaires (as common in web-based trials). Note: In traditional offline trials, an open trial (open-label trial) is a type of clinical trial in which both the researchers and participants know which treatment is being administered. To avoid confusion, use “blinded” or “unblinded” to indicated the level of blinding instead of “open”, as “open” in web-based trials usually refers to “open access” (i.e. participants can self-enrol). (Note: Only report in the abstract what the main paper is reporting. If this information is missing from the main body of text, consider adding it)

	1	2	3	4	5	
subitem not at all important	○	○	○	○	○	essential

Does your paper address subitem 1b-iii?

Copy and paste relevant sections from the manuscript abstract (include quotes in quotation marks "like this" to indicate direct quotes from your manuscript), or elaborate on this item by providing additional information not in the ms, or briefly explain why the item is not applicable/relevant for your study

"Outcomes included knowledge of dementia risk factors, intentions to engage in risk reduction, and health behaviours, measured at baseline, 4 weeks, and 2 months post-intervention."

1b-iv) RESULTS section in abstract must contain use data

Report number of participants enrolled/assessed in each group, the use/uptake of the intervention (e.g., attrition/adherence metrics, use over time, number of logins etc.), in addition to primary/secondary outcomes. (Note: Only report in the abstract what the main paper is reporting. If this information is missing from the main body of text, consider adding it)

	1	2	3	4	5	
subitem not at all important	○	○	○	○	○	essential

Does your paper address subitem 1b-iv?

Copy and paste relevant sections from the manuscript abstract (include quotes in quotation marks "like this" to indicate direct quotes from your manuscript), or elaborate on this item by providing additional information not in the ms, or briefly explain why the item is not applicable/relevant for your study

"A two-arm RCT was conducted with 510 participants (265 in the intervention group and 245 in the control group). "

1b-v) CONCLUSIONS/DISCUSSION in abstract for negative trials

Conclusions/Discussions in abstract for negative trials: Discuss the primary outcome - if the trial is negative (primary outcome not changed), and the intervention was not used, discuss whether negative results are attributable to lack of uptake and discuss reasons. (Note: Only report in the abstract what the main paper is reporting. If this information is missing from the main body of text, consider adding it)

	1	2	3	4	5	
subitem not at all important	○	○	○	○	○	essential

Does your paper address subitem 1b-v?

Copy and paste relevant sections from the manuscript abstract (include quotes in quotation marks "like this" to indicate direct quotes from your manuscript), or elaborate on this item by providing additional information not in the ms, or briefly explain why the item is not applicable/relevant for your study

"An internet-based dementia prevention e-learning program effectively enhanced knowledge and promoted healthy behaviours related to dementia risk reduction. "

INTRODUCTION

2a) In INTRODUCTION: Scientific background and explanation of rationale

2a-i) Problem and the type of system/solution

Describe the problem and the type of system/solution that is object of the study: intended as stand-alone intervention vs. incorporated in broader health care program? Intended for a particular patient population? Goals of the intervention, e.g., being more cost-effective to other interventions, replace or complement other solutions? (Note: Details about the intervention are provided in “Methods” under 5)

	1	2	3	4	5	
subitem not at all important	○	○	○	○	○	essential

Does your paper address subitem 2a-i? *

Copy and paste relevant sections from the manuscript (include quotes in quotation marks "like this" to indicate direct quotes from your manuscript), or elaborate on this item by providing additional information not in the ms, or briefly explain why the item is not applicable/relevant for your study

"Web-based education offers a promising alternative due to its scalability, flexibility, and accessibility. Evidence from other health domains shows that e-learning tools that incorporate multimedia elements, such as audio narration, visuals, and interactive quizzes, are more engaging and improve long-term knowledge retention compared to static, text-only formats (14–16). However, most of the web-based content about modifying dementia risk factors is text-based, such as static web pages or online PDF pamphlets/booklets, often requires a substantial time commitment, is frequently associated with a commercial entity or product for sale, or requires real-time participation (14,17,18). Moreover, few, if any, evidence-based multimedia interventions on this topic have been rigorously studied using randomized controlled trials, particularly those freely accessible to the public in multiple languages. "

2a-ii) Scientific background, rationale: What is known about the (type of) system

Scientific background, rationale: What is known about the (type of) system that is the object of the study (be sure to discuss the use of similar systems for other conditions/diagnoses, if appropiate), motivation for the study, i.e. what are the reasons for and what is the context for this specific study, from which stakeholder viewpoint is the study performed, potential impact of findings [2]. Briefly justify the choice of the comparator.

	1	2	3	4	5	
subitem not at all important	○	○	○	○	○	essential

Does your paper address subitem 2a-ii? *

Copy and paste relevant sections from the manuscript (include quotes in quotation marks "like this" to indicate direct quotes from your manuscript), or elaborate on this item by providing additional information not in the ms, or briefly explain why the item is not applicable/relevant for your study

"Educational interventions have the potential to improve knowledge of dementia risk and promote health behaviour change (11,12). While much of the existing literature has focused on training healthcare providers, a smaller number of studies have examined public education initiatives, which typically involve synchronous, one-on-one delivery by professionals. Most existing resources are predominantly text-based and often lack interactivity or adherence to principles of instructional design (13)."

2b) In INTRODUCTION: Specific objectives or hypotheses

Does your paper address CONSORT subitem 2b? *

Copy and paste relevant sections from the manuscript (include quotes in quotation marks "like this" to indicate direct quotes from your manuscript), or elaborate on this item by providing additional information not in the ms, or briefly explain why the item is not applicable/relevant for your study

"The objectives of this study were to: (1) evaluate whether exposure to an e-learning intervention (DementiaRisk.ca) changes knowledge of dementia risk factors, intention to engage in risk reduction activities, and health behaviours related to dementia risk reduction; and (2) explore qualitative aspects such as participants' engagement and satisfaction with the intervention, as well as barriers and facilitators to use and dissemination of the information provided by the intervention, in adults without cognitive impairment."

METHODS

3a) Description of trial design (such as parallel, factorial) including allocation ratio

Does your paper address CONSORT subitem 3a? *

Copy and paste relevant sections from the manuscript (include quotes in quotation marks "like this" to indicate direct quotes from your manuscript), or elaborate on this item by providing additional information not in the ms, or briefly explain why the item is not applicable/relevant for your study

"This study used a sequential explanatory mixed-methods design, consisting of a two-arm randomized controlled trial (RCT), followed by a qualitative thematic content analysis to explore the findings from the RCT in greater depth. Full details of the methods have been published (19). No changes were made from the published protocol. A mixed-methods design was chosen to incorporate both quantitative and qualitative analysis which allowed for a more comprehensive and nuanced understanding of the relationship between the intervention and its impacts."

3b) Important changes to methods after trial commencement (such as eligibility criteria), with reasons

Does your paper address CONSORT subitem 3b? *

Copy and paste relevant sections from the manuscript (include quotes in quotation marks "like this" to indicate direct quotes from your manuscript), or elaborate on this item by providing additional information not in the ms, or briefly explain why the item is not applicable/relevant for your study

"Full details of the methods have been published (19). No changes were made from the published protocol. "

3b-i) Bug fixes, Downtimes, Content Changes

Bug fixes, Downtimes, Content Changes: ehealth systems are often dynamic systems. A description of changes to methods therefore also includes important changes made on the intervention or comparator during the trial (e.g., major bug fixes or changes in the functionality or content) (5-iii) and other “unexpected events” that may have influenced study design such as staff changes, system failures/downtimes, etc. [2].

	1	2	3	4	5	
subitem not at all important	○	○	○	○	○	essential

Does your paper address subitem 3b-i?

Copy and paste relevant sections from the manuscript (include quotes in quotation marks "like this" to indicate direct quotes from your manuscript), or elaborate on this item by providing additional information not in the ms, or briefly explain why the item is not applicable/relevant for your study

Not applicable for study - there were no bug fixes, downtimes, or content changes.

4a) Eligibility criteria for participants

Does your paper address CONSORT subitem 4a? *

Copy and paste relevant sections from the manuscript (include quotes in quotation marks "like this" to indicate direct quotes from your manuscript), or elaborate on this item by providing additional information not in the ms, or briefly explain why the item is not applicable/relevant for your study

"Participants were recruited from March 2023 to April 2023 using AskingCanadians, a paid panel service to allow for the recruitment of a representative and diverse sample of Canadians. Eligible participants were English-speaking adults with access to email and internet, 18 years of age or older who had never been diagnosed with dementia. "

4a-i) Computer / Internet literacy

Computer / Internet literacy is often an implicit “de facto” eligibility criterion - this should be explicitly clarified.

	1	2	3	4	5	
subitem not at all important	○	○	○	○	○	essential

Does your paper address subitem 4a-i?

Copy and paste relevant sections from the manuscript (include quotes in quotation marks "like this" to indicate direct quotes from your manuscript), or elaborate on this item by providing additional information not in the ms, or briefly explain why the item is not applicable/relevant for your study

"Participants were recruited from March 2023 to April 2023 using AskingCanadians, a paid panel service to allow for the recruitment of a representative and diverse sample of Canadians. Eligible participants were English-speaking adults with access to email and internet, 18 years of age or older who had never been diagnosed with dementia. "

Note: our published and cited protocol paper lists the full eligibility criteria

4a-ii) Open vs. closed, web-based vs. face-to-face assessments:

Open vs. closed, web-based vs. face-to-face assessments: Mention how participants were recruited (online vs. offline), e.g., from an open access website or from a clinic, and clarify if this was a purely web-based trial, or there were face-to-face components (as part of the intervention or for assessment), i.e., to what degree got the study team to know the participant. In online-only trials, clarify if participants were quasi-anonymous and whether having multiple identities was possible or whether technical or logistical measures (e.g., cookies, email confirmation, phone calls) were used to detect/prevent these.

	1	2	3	4	5	
subitem not at all important	○	○	○	○	○	essential

Does your paper address subitem 4a-ii? *

Copy and paste relevant sections from the manuscript (include quotes in quotation marks "like this" to indicate direct quotes from your manuscript), or elaborate on this item by providing additional information not in the ms, or briefly explain why the item is not applicable/relevant for your study

"Participants were recruited from March 2023 to April 2023 using AskingCanadians, a paid panel service to allow for the recruitment of a representative and diverse sample of Canadians."

"After meeting eligibility criteria, participants were redirected to our online research study platform to complete informed consent and baseline measures. Informed consent was obtained digitally, with participants required to review an online information page before consenting to participate."

4a-iii) Information giving during recruitment

Information given during recruitment. Specify how participants were briefed for recruitment and in the informed consent procedures (e.g., publish the informed consent documentation as appendix, see also item X26), as this information may have an effect on user self-selection, user expectation and may also bias results.

	1	2	3	4	5	
subitem not at all important	○	○	○	○	○	essential

Does your paper address subitem 4a-iii?

Copy and paste relevant sections from the manuscript (include quotes in quotation marks "like this" to indicate direct quotes from your manuscript), or elaborate on this item by providing additional information not in the ms, or briefly explain why the item is not applicable/relevant for your study

"Participants were recruited from March 2023 to April 2023 using AskingCanadians, a paid panel service to allow for the recruitment of a representative and diverse sample of Canadians."

"After meeting eligibility criteria, participants were redirected to our online research study platform to complete informed consent and baseline measures. Informed consent was obtained digitally, with participants required to review an online information page before consenting to participate."

4b) Settings and locations where the data were collected

Does your paper address CONSORT subitem 4b? *

Copy and paste relevant sections from the manuscript (include quotes in quotation marks "like this" to indicate direct quotes from your manuscript), or elaborate on this item by providing additional information not in the ms, or briefly explain why the item is not applicable/relevant for your study

"After meeting eligibility criteria, participants were redirected to our online research study platform to complete informed consent and baseline measures. Informed consent was obtained digitally, with participants required to review an online information page before consenting to participate."

4b-i) Report if outcomes were (self-)assessed through online questionnaires

Clearly report if outcomes were (self-)assessed through online questionnaires (as common in web-based trials) or otherwise.

	1	2	3	4	5	
subitem not at all important	○	○	○	○	○	essential

Does your paper address subitem 4b-i? *

Copy and paste relevant sections from the manuscript (include quotes in quotation marks "like this" to indicate direct quotes from your manuscript), or elaborate on this item by providing additional information not in the ms, or briefly explain why the item is not applicable/relevant for your study

"Quantitative data was collected through online questionnaires with established reliability and validity at baseline (T1), 4-weeks (T2), and at 2-months post-intervention (T3). "

"All data were collected through secure password-protected online platforms, which automatically recorded responses and stored data. Participant responses were anonymized prior to analysis. Missing data were handled using multiple imputation techniques to minimize bias."

4b-ii) Report how institutional affiliations are displayed

Report how institutional affiliations are displayed to potential participants [on ehealth media], as affiliations with prestigious hospitals or universities may affect volunteer rates, use, and reactions with regards to an intervention.(Not a required item – describe only if this may bias results)

	1	2	3	4	5	
subitem not at all important	○	○	○	○	○	essential

Does your paper address subitem 4b-ii?

Copy and paste relevant sections from the manuscript (include quotes in quotation marks "like this" to indicate direct quotes from your manuscript), or elaborate on this item by providing additional information not in the ms, or briefly explain why the item is not applicable/relevant for your study

"Ethical approval was provided by the Hamilton Integrated Research Ethics Board (Project ID 14886). "

5) The interventions for each group with sufficient details to allow replication, including how and when they were actually administered

5-i) Mention names, credential, affiliations of the developers, sponsors, and owners

Mention names, credential, affiliations of the developers, sponsors, and owners [6] (if authors/evaluators are owners or developer of the software, this needs to be declared in a “Conflict of interest” section or mentioned elsewhere in the manuscript).

	1	2	3	4	5	
subitem not at all important	○	○	○	○	○	essential

Does your paper address subitem 5-i?

Copy and paste relevant sections from the manuscript (include quotes in quotation marks "like this" to indicate direct quotes from your manuscript), or elaborate on this item by providing additional information not in the ms, or briefly explain why the item is not applicable/relevant for your study

"The intervention group received online access to a 35-minute multimedia e-learning lesson (DementiaRisk.ca) about dementia risk and protective factors (Figure 1), as well as a series of 12 pre-scheduled micro-learning emails. The emails included small, focused segments of content designed to reinforce key information from the e-learning lesson through spaced repetition.

Participants had four weeks to complete the e-learning lesson and received three emails per week during that period.

Content for the intervention was based predominantly on the World Health Organization Guidelines on risk reduction for cognitive decline and dementia and the Lancet Commission's 2020 report on dementia prevention, intervention, and care (4,20). Instructional design was guided by Mayer's Cognitive Theory of Multimedia Learning, ensuring that materials were delivered in manageable segments, using a combination of visuals, audio narration, and other evidence-based principles essential to effective e-learning that have been shown to enhance knowledge retention (13,14,16,17). "

"The control group participants received online access to a 35-minute lesson on mild cognitive impairment (MCI) (Figure 2) and a series of 12 micro-learning emails with small segments of content to reinforce material from the lesson. The design of the control group e-learning was also informed by similar principles and theoretical frameworks. "

5-ii) Describe the history/development process

Describe the history/development process of the application and previous formative evaluations (e.g., focus groups, usability testing), as these will have an impact on adoption/use rates and help with interpreting results.

	1	2	3	4	5	
subitem not at all important	○	○	○	○	○	essential

Does your paper address subitem 5-ii?

Copy and paste relevant sections from the manuscript (include quotes in quotation marks "like this" to indicate direct quotes from your manuscript), or elaborate on this item by providing additional information not in the ms, or briefly explain why the item is not applicable/relevant for your study

"Full details of the methods have been published (19). No changes were made from the published protocol."

5-iii) Revisions and updating

Revisions and updating. Clearly mention the date and/or version number of the application/intervention (and comparator, if applicable) evaluated, or describe whether the intervention underwent major changes during the evaluation process, or whether the development and/or content was "frozen" during the trial. Describe dynamic components such as news feeds or changing content which may have an impact on the replicability of the intervention (for unexpected events see item 3b).

	1	2	3	4	5	
subitem not at all important	○	○	○	○	○	essential

Does your paper address subitem 5-iii?

Copy and paste relevant sections from the manuscript (include quotes in quotation marks "like this" to indicate direct quotes from your manuscript), or elaborate on this item by providing additional information not in the ms, or briefly explain why the item is not applicable/relevant for your study

"Full details of the methods have been published (19). No changes were made from the published protocol."

5-iv) Quality assurance methods

Provide information on quality assurance methods to ensure accuracy and quality of information provided [1], if applicable.

	1	2	3	4	5	
subitem not at all important	○	○	○	○	○	essential

Does your paper address subitem 5-iv?

Copy and paste relevant sections from the manuscript (include quotes in quotation marks "like this" to indicate direct quotes from your manuscript), or elaborate on this item by providing additional information not in the ms, or briefly explain why the item is not applicable/relevant for your study

N/A

Your answer must have a minimum of 25 characters.

5-v) Ensure replicability by publishing the source code, and/or providing screenshots/screen-capture video, and/or providing flowcharts of the algorithms used

Ensure replicability by publishing the source code, and/or providing screenshots/screen-capture video, and/or providing flowcharts of the algorithms used. Replicability (i.e., other researchers should in principle be able to replicate the study) is a hallmark of scientific reporting.

	1	2	3	4	5	
subitem not at all important	○	○	○	○	○	essential

Does your paper address subitem 5-v?

Copy and paste relevant sections from the manuscript (include quotes in quotation marks "like this" to indicate direct quotes from your manuscript), or elaborate on this item by providing additional information not in the ms, or briefly explain why the item is not applicable/relevant for your study

N/A (no source code/algorithms used).

5-vi) Digital preservation

Digital preservation: Provide the URL of the application, but as the intervention is likely to change or disappear over the course of the years; also make sure the intervention is archived (Internet Archive, webcitation.org, and/or publishing the source code or screenshots/videos alongside the article). As pages behind login screens cannot be archived, consider creating demo pages which are accessible without login.

	1	2	3	4	5	
subitem not at all important	○	○	○	○	○	essential

Does your paper address subitem 5-vi?

Copy and paste relevant sections from the manuscript (include quotes in quotation marks "like this" to indicate direct quotes from your manuscript), or elaborate on this item by providing additional information not in the ms, or briefly explain why the item is not applicable/relevant for your study

https://dementiarisk.ca
https://www.mcmasteroptimalaging.org/e-learning/dementia-risk

5-vii) Access

Access: Describe how participants accessed the application, in what setting/context, if they had to pay (or were paid) or not, whether they had to be a member of specific group. If known, describe how participants obtained "access to the platform and Internet" [1]. To ensure access for editors/reviewers/readers, consider to provide a "backdoor" login account or demo mode for reviewers/readers to explore the application (also important for archiving purposes, see vi).

	1	2	3	4	5	
subitem not at all important	○	○	○	○	○	essential

Does your paper address subitem 5-vii? *

Copy and paste relevant sections from the manuscript (include quotes in quotation marks "like this" to indicate direct quotes from your manuscript), or elaborate on this item by providing additional information not in the ms, or briefly explain why the item is not applicable/relevant for your study

"Participants were recruited from March 2023 to April 2023 using AskingCanadians, a paid panel service to allow for the recruitment of a representative and diverse sample of Canadians."

"The intervention group received online access to a 35-minute multimedia e-learning lesson (DementiaRisk.ca) about dementia risk and protective factors (Figure 1), as well as a series of 12 pre-scheduled micro-learning emails. "

"The control group participants received online access to a 35-minute lesson on mild cognitive impairment (MCI) (Figure 2) and a series of 12 micro-learning emails with small segments of content to reinforce material from the lesson."

5-viii) Mode of delivery, features/functionalities/components of the intervention and comparator, and the theoretical framework

Describe mode of delivery, features/functionalities/components of the intervention and comparator, and the theoretical framework [6] used to design them (instructional strategy [1], behaviour change techniques, persuasive features, etc., see e.g., [7, 8] for terminology). This includes an in-depth description of the content (including where it is coming from and who developed it) [1],” whether [and how] it is tailored to individual circumstances and allows users to track their progress and receive feedback” [6]. This also includes a description of communication delivery channels and – if computer-mediated communication is a component – whether communication was synchronous or asynchronous [6]. It also includes information on presentation strategies [1], including page design principles, average amount of text on pages, presence of hyperlinks to other resources, etc. [1].

	1	2	3	4	5	
subitem not at all important	○	○	○	○	○	essential

Does your paper address subitem 5-viii? *

Copy and paste relevant sections from the manuscript (include quotes in quotation marks "like this" to indicate direct quotes from your manuscript), or elaborate on this item by providing additional information not in the ms, or briefly explain why the item is not applicable/relevant for your study

"Other theoretical frameworks also informed the overall design of the intervention and its evaluation. The Theory of Planned Behaviour (TPB) (21,22) was used due to its extensive application with a number of dementia prevention risk factors (e.g., smoking, diet, alcohol consumption, and physical activity) (23–27). TPB suggests that intentions to engage in or change specific behaviours depend on motivation and ability across six constructs that collectively represent a person’s actual control over their behaviour. DementiaRisk aimed to change participants’ attitudes and behavioural intentions by increasing knowledge related to dementia prevention and modifiable risk factors. The intervention and its evaluation were also informed by the Acquisition Cognition Application – Level of Outcomes model, which helps explain the value of the information from the user perspective, looking at the domains of situational relevance, cognitive impact, use of the information, and if used, any perceived health benefits (28). "

5-ix) Describe use parameters

Describe use parameters (e.g., intended “doses” and optimal timing for use). Clarify what instructions or recommendations were given to the user, e.g., regarding timing, frequency, heaviness of use, if any, or was the intervention used ad libitum.

	1	2	3	4	5	
subitem not at all important	○	○	○	○	○	essential

Does your paper address subitem 5-ix?

Copy and paste relevant sections from the manuscript (include quotes in quotation marks "like this" to indicate direct quotes from your manuscript), or elaborate on this item by providing additional information not in the ms, or briefly explain why the item is not applicable/relevant for your study

"Participants had four weeks to complete the e-learning lesson and received three emails per week during that period. "

5-x) Clarify the level of human involvement

Clarify the level of human involvement (care providers or health professionals, also technical assistance) in the e-intervention or as co-intervention (detail number and expertise of professionals involved, if any, as well as “type of assistance offered, the timing and frequency of the support, how it is initiated, and the medium by which the assistance is delivered”. It may be necessary to distinguish between the level of human involvement required for the trial, and the level of human involvement required for a routine application outside of a RCT setting (discuss under item 21 – generalizability).

	1	2	3	4	5	
subitem not at all important	○	○	○	○	○	essential

Does your paper address subitem 5-x?

Copy and paste relevant sections from the manuscript (include quotes in quotation marks "like this" to indicate direct quotes from your manuscript), or elaborate on this item by providing additional information not in the ms, or briefly explain why the item is not applicable/relevant for your study

All components were delivered online, there was no human involvement.

5-xi) Report any prompts/reminders used

Report any prompts/reminders used: Clarify if there were prompts (letters, emails, phone calls, SMS) to use the application, what triggered them, frequency etc. It may be necessary to distinguish between the level of prompts/reminders required for the trial, and the level of prompts/reminders for a routine application outside of a RCT setting (discuss under item 21 – generalizability).

	1	2	3	4	5	
subitem not at all important	○	○	○	○	○	essential

Does your paper address subitem 5-xi? *

Copy and paste relevant sections from the manuscript (include quotes in quotation marks "like this" to indicate direct quotes from your manuscript), or elaborate on this item by providing additional information not in the ms, or briefly explain why the item is not applicable/relevant for your study

"Participants had four weeks to complete the e-learning lesson and received three emails per week during that period. "

5-xii) Describe any co-interventions (incl. training/support)

Describe any co-interventions (incl. training/support): Clearly state any interventions that are provided in addition to the targeted eHealth intervention, as ehealth intervention may not be designed as stand-alone intervention. This includes training sessions and support [1]. It may be necessary to distinguish between the level of training required for the trial, and the level of training for a routine application outside of a RCT setting (discuss under item 21 – generalizability.

	1	2	3	4	5	
subitem not at all important	○	○	○	○	○	essential

Does your paper address subitem 5-xii? *

Copy and paste relevant sections from the manuscript (include quotes in quotation marks "like this" to indicate direct quotes from your manuscript), or elaborate on this item by providing additional information not in the ms, or briefly explain why the item is not applicable/relevant for your study

This is not applicable; this paper and our referenced protocol paper describes our intervention in full.

6a) Completely defined pre-specified primary and secondary outcome measures, including how and when they were assessed

Does your paper address CONSORT subitem 6a? *

Copy and paste relevant sections from the manuscript (include quotes in quotation marks "like this" to indicate direct quotes from your manuscript), or elaborate on this item by providing additional information not in the ms, or briefly explain why the item is not applicable/relevant for your study

"Quantitative data was collected through online questionnaires with established reliability and validity at baseline (T1), 4-weeks (T2), and at 2-months post-intervention (T3). Knowledge of dementia risk factors was collected using a custom 15-item multiple choice assessment aligned with the content of the intervention as well as the Risks and Health Promotion sub-domain of the Dementia Knowledge Assessment Scale (DKAS) (29). Intentions to engage in health behaviours were measured using a custom 10-item assessment. Current participant health behaviours related to dementia risk factors were assessed using the Godin-Shephard Leisure-Time Physical Activity Questionnaire (the Godin) for physical activity (30) along with additional questions related to diet, smoking, alcohol consumption, social activity, traumatic brain injury (TBI), blood pressure, depression, air pollution, diabetes, sleep habits, brain activity, and hearing. "

"Data related to engagement with the e-learning programs were collected during the active study phase to gain a clearer understanding of how the use of web-based learning throughout the study (or fidelity of delivery) might have impacted changes in knowledge, intentions, or health behaviours. The following metrics were used: number of e-learning starts and completions, e-learning completion rates, time spent on lessons, and micro-learning email open rates. "

6a-i) Online questionnaires: describe if they were validated for online use and apply CHERRIES items to describe how the questionnaires were designed/deployed

If outcomes were obtained through online questionnaires, describe if they were validated for online use and apply CHERRIES items to describe how the questionnaires were designed/deployed [9].

	1	2	3	4	5	
subitem not at all important	○	○	○	○	○	essential

Does your paper address subitem 6a-i?

Copy and paste relevant sections from manuscript text

Yes, the CHERRIES checklist has been included as a supplemental file.

6a-ii) Describe whether and how “use” (including intensity of use/dosage) was defined/measured/monitored

Describe whether and how “use” (including intensity of use/dosage) was defined/measured/monitored (logins, logfile analysis, etc.). Use/adoption metrics are important process outcomes that should be reported in any ehealth trial.

	1	2	3	4	5	
subitem not at all important	○	○	○	○	○	essential

Does your paper address subitem 6a-ii?

Copy and paste relevant sections from manuscript text

"After meeting eligibility criteria, participants were redirected to our online research study platform to complete informed consent and baseline measures. "

"Following completion of online informed consent, participants were randomized and directed to their assigned intervention or control group learning path. "

6a-iii) Describe whether, how, and when qualitative feedback from participants was obtained

Describe whether, how, and when qualitative feedback from participants was obtained (e.g., through emails, feedback forms, interviews, focus groups).

	1	2	3	4	5	
subitem not at all important	○	○	○	○	○	essential

Does your paper address subitem 6a-iii?

Copy and paste relevant sections from manuscript text

"Qualitative data were collected from participants through both structured and open-ended questions using the Information Assessment Method for patients and consumers (IAM4all), a validated questionnaire that assesses outcomes of online consumer health information (31). Qualitative questions explored participant satisfaction with the intervention, changes in attitudes, beliefs, and behaviours during the study, and feedback on proposed future dissemination methods."

6b) Any changes to trial outcomes after the trial commenced, with reasons

Does your paper address CONSORT subitem 6b? *

Copy and paste relevant sections from the manuscript (include quotes in quotation marks "like this" to indicate direct quotes from your manuscript), or elaborate on this item by providing additional information not in the ms, or briefly explain why the item is not applicable/relevant for your study

Not applicable, there were no changes to trial outcomes after the trial commenced.

7a) How sample size was determined

NPT: When applicable, details of whether and how the clustering by care provides or centers was addressed

7a-i) Describe whether and how expected attrition was taken into account when calculating the sample size

Describe whether and how expected attrition was taken into account when calculating the sample size.

	1	2	3	4	5	
subitem not at all important	○	○	○	○	○	essential

Does your paper address subitem 7a-i?

Copy and paste relevant sections from manuscript title (include quotes in quotation marks "like this" to indicate direct quotes from your manuscript), or elaborate on this item by providing additional information not in the ms, or briefly explain why the item is not applicable/relevant for your study

"Using a conservative estimate of a small effect size (0.16, from a meta-analysis of internet health behaviour change interventions), with a power of 0.80 and alpha of 0.05, we aimed to recruit a total of 388 participants in the study (36,37). The sample size was increased by 25% to account for anticipated dropout, which brought the target recruitment number to 485 individuals (36,37). These recruitment numbers and strategies have been used successfully in similar knowledge translation intervention studies and were a feasible target given the use of the paid panel (38)."

7b) When applicable, explanation of any interim analyses and stopping guidelines

Does your paper address CONSORT subitem 7b? *

Copy and paste relevant sections from the manuscript (include quotes in quotation marks "like this" to indicate direct quotes from your manuscript), or elaborate on this item by providing additional information not in the ms, or briefly explain why the item is not applicable/relevant for your study

"RStudio was used for quantitative data analysis (32–35). A linear mixed-effects model was used to examine the relationship between each assessment's total score and group by timepoint. Outcome scores from all three timepoints, baseline (T1), at 4 weeks (T2), and 2 months post-intervention (T3), were included as the dependent variable. "

8a) Method used to generate the random allocation sequence

NPT: When applicable, how care providers were allocated to each trial group

Does your paper address CONSORT subitem 8a? *

Copy and paste relevant sections from the manuscript (include quotes in quotation marks "like this" to indicate direct quotes from your manuscript), or elaborate on this item by providing additional information not in the ms, or briefly explain why the item is not applicable/relevant for your study

"Following completion of online informed consent, participants were randomized and directed to their assigned intervention or control group learning path. Participants were randomized in a 1:1 ratio via a computer-generated sequence through our research platform using a permuted block stratified randomization, with education and age as the stratification variables.

To the best of our ability, efforts were made to blind participants to the study hypothesis and allocation group by referring to the study in broad terms, such as 'e-learning about cognitive impairment and promoting brain health' (rather than explicitly 'dementia prevention', for example). The principal investigator [AJL] and data analysts [DH and AS] remained blinded to group allocation throughout the data collection and analysis phases."

8b) Type of randomisation; details of any restriction (such as blocking and block size)

Does your paper address CONSORT subitem 8b? *

Copy and paste relevant sections from the manuscript (include quotes in quotation marks "like this" to indicate direct quotes from your manuscript), or elaborate on this item by providing additional information not in the ms, or briefly explain why the item is not applicable/relevant for your study

"Following completion of online informed consent, participants were randomized and directed to their assigned intervention or control group learning path. Participants were randomized in a 1:1 ratio via a computer-generated sequence through our research platform using a permuted block stratified randomization, with education and age as the stratification variables.

To the best of our ability, efforts were made to blind participants to the study hypothesis and allocation group by referring to the study in broad terms, such as 'e-learning about cognitive impairment and promoting brain health' (rather than explicitly 'dementia prevention', for example). The principal investigator [AJL] and data analysts [DH and AS] remained blinded to group allocation throughout the data collection and analysis phases."

9) Mechanism used to implement the random allocation sequence (such as sequentially numbered containers), describing any steps taken to conceal the sequence until interventions were assigned

Does your paper address CONSORT subitem 9? *

Copy and paste relevant sections from the manuscript (include quotes in quotation marks "like this" to indicate direct quotes from your manuscript), or elaborate on this item by providing additional information not in the ms, or briefly explain why the item is not applicable/relevant for your study

"Following completion of online informed consent, participants were randomized and directed to their assigned intervention or control group learning path. Participants were randomized in a 1:1 ratio via a computer-generated sequence through our research platform using a permuted block stratified randomization, with education and age as the stratification variables.

To the best of our ability, efforts were made to blind participants to the study hypothesis and allocation group by referring to the study in broad terms, such as ‘e-learning about cognitive impairment and promoting brain health’ (rather than explicitly ‘dementia prevention’, for example). The principal investigator [AJL] and data analysts [DH and AS] remained blinded to group allocation throughout the data collection and analysis phases."

10) Who generated the random allocation sequence, who enrolled participants, and who assigned participants to interventions

Does your paper address CONSORT subitem 10? *

Copy and paste relevant sections from the manuscript (include quotes in quotation marks "like this" to indicate direct quotes from your manuscript), or elaborate on this item by providing additional information not in the ms, or briefly explain why the item is not applicable/relevant for your study

"Following completion of online informed consent, participants were randomized and directed to their assigned intervention or control group learning path. Participants were randomized in a 1:1 ratio via a computer-generated sequence through our research platform using a permuted block stratified randomization, with education and age as the stratification variables.

To the best of our ability, efforts were made to blind participants to the study hypothesis and allocation group by referring to the study in broad terms, such as 'e-learning about cognitive impairment and promoting brain health' (rather than explicitly 'dementia prevention', for example). The principal investigator [AJL] and data analysts [DH and AS] remained blinded to group allocation throughout the data collection and analysis phases."

11a) If done, who was blinded after assignment to interventions (for example, participants, care providers, those assessing outcomes) and how

NPT: Whether or not administering co-interventions were blinded to group assignment

11a-i) Specify who was blinded, and who wasn't

Specify who was blinded, and who wasn't. Usually, in web-based trials it is not possible to blind the participants [1, 3] (this should be clearly acknowledged), but it may be possible to blind outcome assessors, those doing data analysis or those administering co-interventions (if any).

	1	2	3	4	5	
subitem not at all important	○	○	○	○	○	essential

Does your paper address subitem 11a-i? *

Copy and paste relevant sections from the manuscript (include quotes in quotation marks "like this" to indicate direct quotes from your manuscript), or elaborate on this item by providing additional information not in the ms, or briefly explain why the item is not applicable/relevant for your study

"To the best of our ability, efforts were made to blind participants to the study hypothesis and allocation group by referring to the study in broad terms, such as 'e-learning about cognitive impairment and promoting brain health' (rather than explicitly 'dementia prevention', for example). The principal investigator [AJL] and data analysts [DH and AS] remained blinded to group allocation throughout the data collection and analysis phases."

11a-ii) Discuss e.g., whether participants knew which intervention was the "intervention of interest" and which one was the "comparator"

Informed consent procedures (4a-ii) can create biases and certain expectations - discuss e.g., whether participants knew which intervention was the "intervention of interest" and which one was the "comparator".

	1	2	3	4	5	
subitem not at all important	○	○	○	○	○	essential

Does your paper address subitem 11a-ii?

Copy and paste relevant sections from the manuscript (include quotes in quotation marks "like this" to indicate direct quotes from your manuscript), or elaborate on this item by providing additional information not in the ms, or briefly explain why the item is not applicable/relevant for your study

"To the best of our ability, efforts were made to blind participants to the study hypothesis and allocation group by referring to the study in broad terms, such as 'e-learning about cognitive impairment and promoting brain health' (rather than explicitly 'dementia prevention', for example). The principal investigator [AJL] and data analysts [DH and AS] remained blinded to group allocation throughout the data collection and analysis phases."

11b) If relevant, description of the similarity of interventions
(this item is usually not relevant for ehealth trials as it refers to similarity of a placebo or sham intervention to a active medication/intervention)

Does your paper address CONSORT subitem 11b? *

Copy and paste relevant sections from the manuscript (include quotes in quotation marks "like this" to indicate direct quotes from your manuscript), or elaborate on this item by providing additional information not in the ms, or briefly explain why the item is not applicable/relevant for your study

Not applicable for e-health trials as stated

12a) Statistical methods used to compare groups for primary and secondary outcomes
NPT: When applicable, details of whether and how the clustering by care providers or centers was addressed

Does your paper address CONSORT subitem 12a? *

Copy and paste relevant sections from the manuscript (include quotes in quotation marks "like this" to indicate direct quotes from your manuscript), or elaborate on this item by providing additional information not in the ms, or briefly explain why the item is not applicable/relevant for your study

"RStudio was used for quantitative data analysis (32–35). A linear mixed-effects model was used to examine the relationship between each assessment's total score and group by timepoint. Outcome scores from all three timepoints, baseline (T1), at 4 weeks (T2), and 2 months post-intervention (T3), were included as the dependent variable. Participant ID was included as a random effect, and an interaction between time (factor variable, T1, T2, and T3) and group was used to examine the possibility of effect modification by group over time on the outcome. Gender, as well as both stratification factors – age group (<45, 45-64, [illegible] 65) and education (high school equivalent or less, some college/university, college/university or graduate degree) – were included in all models."

12a-i) Imputation techniques to deal with attrition / missing values

Imputation techniques to deal with attrition / missing values: Not all participants will use the intervention/comparator as intended and attrition is typically high in ehealth trials. Specify how participants who did not use the application or dropped out from the trial were treated in the statistical analysis (a complete case analysis is strongly discouraged, and simple imputation techniques such as LOCF may also be problematic [4]).

	1	2	3	4	5	
subitem not at all important	○	○	○	○	○	essential

Does your paper address subitem 12a-i? *

Copy and paste relevant sections from the manuscript (include quotes in quotation marks "like this" to indicate direct quotes from your manuscript), or elaborate on this item by providing additional information not in the ms, or briefly explain why the item is not applicable/relevant for your study

"All data were collected through secure password-protected online platforms, which automatically recorded responses and stored data. Participant responses were anonymized prior to analysis. Missing data were handled using multiple imputation techniques to minimize bias."

12b) Methods for additional analyses, such as subgroup analyses and adjusted analyses

Does your paper address CONSORT subitem 12b? *

Copy and paste relevant sections from the manuscript (include quotes in quotation marks "like this" to indicate direct quotes from your manuscript), or elaborate on this item by providing additional information not in the ms, or briefly explain why the item is not applicable/relevant for your study

"RStudio was used for quantitative data analysis (32–35). A linear mixed-effects model was used to examine the relationship between each assessment’s total score and group by timepoint. Outcome scores from all three timepoints, baseline (T1), at 4 weeks (T2), and 2 months post-intervention (T3), were included as the dependent variable. Participant ID was included as a random effect, and an interaction between time (factor variable, T1, T2, and T3) and group was used to examine the possibility of effect modification by group over time on the outcome. Gender, as well as both stratification factors – age group (<45, 45-64, [illegible] 65) and education (high school equivalent or less, some college/university, college/university or graduate degree) – were included in all models."

X26) REB/IRB Approval and Ethical Considerations [recommended as subheading under "Methods"] (not a CONSORT item)

X26-i) Comment on ethics committee approval

	1	2	3	4	5	
subitem not at all important	○	○	○	○	○	essential

Does your paper address subitem X26-i?

Copy and paste relevant sections from the manuscript (include quotes in quotation marks "like this" to indicate direct quotes from your manuscript), or elaborate on this item by providing additional information not in the ms, or briefly explain why the item is not applicable/relevant for your study

"Ethical approval was provided by the Hamilton Integrated Research Ethics Board (Project ID 14886). "

x26-ii) Outline informed consent procedures

Outline informed consent procedures e.g., if consent was obtained offline or online (how? Checkbox, etc.?), and what information was provided (see 4a-ii). See [6] for some items to be included in informed consent documents.

	1	2	3	4	5	
subitem not at all important	○	○	○	○	○	essential

Does your paper address subitem X26-ii?

Copy and paste relevant sections from the manuscript (include quotes in quotation marks "like this" to indicate direct quotes from your manuscript), or elaborate on this item by providing additional information not in the ms, or briefly explain why the item is not applicable/relevant for your study

"After meeting eligibility criteria, participants were redirected to our online research study platform to complete informed consent and baseline measures. Informed consent was obtained digitally, with participants required to review an online information page before consenting to participate. "

X26-iii) Safety and security procedures

Safety and security procedures, incl. privacy considerations, and any steps taken to reduce the likelihood or detection of harm (e.g., education and training, availability of a hotline)

	1	2	3	4	5	
subitem not at all important	○	○	○	○	○	essential

Does your paper address subitem X26-iii?

Copy and paste relevant sections from the manuscript (include quotes in quotation marks "like this" to indicate direct quotes from your manuscript), or elaborate on this item by providing additional information not in the ms, or briefly explain why the item is not applicable/relevant for your study

Not applicable; no likelihood of harm.

RESULTS

13a) For each group, the numbers of participants who were randomly assigned, received intended treatment, and were analysed for the primary outcome

NPT: The number of care providers or centers performing the intervention in each group and the number of patients treated by each care provider in each center

Does your paper address CONSORT subitem 13a? *

Copy and paste relevant sections from the manuscript (include quotes in quotation marks "like this" to indicate direct quotes from your manuscript), or elaborate on this item by providing additional information not in the ms, or briefly explain why the item is not applicable/relevant for your study

"A total of 1319 individuals responded to the initial online recruitment message and 879 eligible participants were invited to complete informed consent. 534 individuals consented and were randomly assigned to the intervention (n = 272) or control group (n = 262). 510 participants began the study (intervention n=265; control n=245). Retention at T3 was 79.4% (intervention n=211; control n=194). "

13b) For each group, losses and exclusions after randomisation, together with reasons

Does your paper address CONSORT subitem 13b? (NOTE: Preferably, this is shown in a CONSORT flow diagram) *

Copy and paste relevant sections from the manuscript (include quotes in quotation marks "like this" to indicate direct quotes from your manuscript), or elaborate on this item by providing additional information not in the ms, or briefly explain why the item is not applicable/relevant for your study

"A total of 1319 individuals responded to the initial online recruitment message and 879 eligible participants were invited to complete informed consent. 534 individuals consented and were randomly assigned to the intervention (n = 272) or control group (n = 262). 510 participants began the study (intervention n=265; control n=245). Retention at T3 was 79.4% (intervention n=211; control n=194). "

13b-i) Attrition diagram

Strongly recommended: An attrition diagram (e.g., proportion of participants still logging in or using the intervention/comparator in each group plotted over time, similar to a survival curve) or other figures or tables demonstrating usage/dose/engagement.

	1	2	3	4	5	
subitem not at all important	○	○	○	○	○	essential

Does your paper address subitem 13b-i?

Copy and paste relevant sections from the manuscript or cite the figure number if applicable (include quotes in quotation marks "like this" to indicate direct quotes from your manuscript), or elaborate on this item by providing additional information not in the ms, or briefly explain why the item is not applicable/relevant for your study

"A total of 1319 individuals responded to the initial online recruitment message and 879 eligible participants were invited to complete informed consent. 534 individuals consented and were randomly assigned to the intervention (n = 272) or control group (n = 262). 510 participants began the study (intervention n=265; control n=245). Retention at T3 was 79.4% (intervention n=211; control n=194). "

CONSORT Flow is also included as an attachment

14a) Dates defining the periods of recruitment and follow-up

Does your paper address CONSORT subitem 14a? *

Copy and paste relevant sections from the manuscript (include quotes in quotation marks "like this" to indicate direct quotes from your manuscript), or elaborate on this item by providing additional information not in the ms, or briefly explain why the item is not applicable/relevant for your study

"Participants were recruited from March 2023 to April 2023 using AskingCanadians, a paid panel service to allow for the recruitment of a representative and diverse sample of Canadians. "

14a-i) Indicate if critical "secular events" fell into the study period

Indicate if critical "secular events" fell into the study period, e.g., significant changes in Internet resources available or "changes in computer hardware or Internet delivery resources"

	1	2	3	4	5	
subitem not at all important	○	○	○	○	○	essential

Does your paper address subitem 14a-i?

Copy and paste relevant sections from the manuscript (include quotes in quotation marks "like this" to indicate direct quotes from your manuscript), or elaborate on this item by providing additional information not in the ms, or briefly explain why the item is not applicable/relevant for your study

Not applicable, no events to report on.

14b) Why the trial ended or was stopped (early)

Does your paper address CONSORT subitem 14b? *

Copy and paste relevant sections from the manuscript (include quotes in quotation marks "like this" to indicate direct quotes from your manuscript), or elaborate on this item by providing additional information not in the ms, or briefly explain why the item is not applicable/relevant for your study

Not applicable, the trial was not ended early.

15) A table showing baseline demographic and clinical characteristics for each group

NPT: When applicable, a description of care providers (case volume, qualification, expertise, etc.) and centers (volume) in each group

Does your paper address CONSORT subitem 15? *

Copy and paste relevant sections from the manuscript (include quotes in quotation marks "like this" to indicate direct quotes from your manuscript), or elaborate on this item by providing additional information not in the ms, or briefly explain why the item is not applicable/relevant for your study

"There were no significant differences in baseline characteristics between the intervention and control groups (all p > 0.05). Table 1 presents the demographic details of participants in each group. Participants were predominantly older (54.7% [illegible] 55 or older), female (60.6%), and in good, very good, or excellent health (81.2%). The majority of participants identified as Caucasian (55.3%) followed by Chinese (10.4%) and South Asian or Multiracial (each at 6.1%). Distribution patterns were relatively consistent across groups, though the control group had a slightly higher proportion of multiracial and Indigenous/First Nations participants. When asked about a family history of dementia, 31.4% of participants reported having a family history, 51.6% reported no family history, and 16.1% were unsure. The responses were consistent between groups. Finally, approximately one-quarter of participants (25.1%) identified as being a dementia care partner, with a higher proportion in the intervention group (27.9%) compared to the control group (22.0%)."

15-i) Report demographics associated with digital divide issues

In ehealth trials it is particularly important to report demographics associated with digital divide issues, such as age, education, gender, social-economic status, computer/Internet/ehealth literacy of the participants, if known.

	1	2	3	4	5	
subitem not at all important	○	○	○	○	○	essential

Does your paper address subitem 15-i? *

Copy and paste relevant sections from the manuscript (include quotes in quotation marks "like this" to indicate direct quotes from your manuscript), or elaborate on this item by providing additional information not in the ms, or briefly explain why the item is not applicable/relevant for your study

"There were no significant differences in baseline characteristics between the intervention and control groups (all $p > 0.05$). Table 1 presents the demographic details of participants in each group. Participants were predominantly older (54.7% □ 55 or older), female (60.6%), and in good, very good, or excellent health (81.2%). The majority of participants identified as Caucasian (55.3%) followed by Chinese (10.4%) and South Asian or Multiracial (each at 6.1%). Distribution patterns were relatively consistent across groups, though the control group had a slightly higher proportion of multiracial and Indigenous/First Nations participants. When asked about a family history of dementia, 31.4% of participants reported having a family history, 51.6% reported no family history, and 16.1% were unsure. The responses were consistent between groups. Finally, approximately one-quarter of participants (25.1%) identified as being a dementia care partner, with a higher proportion in the intervention group (27.9%) compared to the control group (22.0%)."

16) For each group, number of participants (denominator) included in each analysis and whether the analysis was by original assigned groups

16-i) Report multiple “denominators” and provide definitions

Report multiple “denominators” and provide definitions: Report N’s (and effect sizes) “across a range of study participation [and use] thresholds” [1], e.g., N exposed, N consented, N used more than x times, N used more than y weeks, N participants “used” the intervention/comparator at specific pre-defined time points of interest (in absolute and relative numbers per group). Always clearly define “use” of the intervention.

	1	2	3	4	5	
subitem not at all important	○	○	○	○	○	essential

Does your paper address subitem 16-i? *

Copy and paste relevant sections from the manuscript (include quotes in quotation marks "like this" to indicate direct quotes from your manuscript), or elaborate on this item by providing additional information not in the ms, or briefly explain why the item is not applicable/relevant for your study

Specific denominators are included in the table (not pasted here)

"There were no significant differences in baseline characteristics between the intervention and control groups (all p > 0.05). Table 1 presents the demographic details of participants in each group. Participants were predominantly older (54.7% [illegible] 55 or older), female (60.6%), and in good, very good, or excellent health (81.2%). The majority of participants identified as Caucasian (55.3%) followed by Chinese (10.4%) and South Asian or Multiracial (each at 6.1%). Distribution patterns were relatively consistent across groups, though the control group had a slightly higher proportion of multiracial and Indigenous/First Nations participants. When asked about a family history of dementia, 31.4% of participants reported having a family history, 51.6% reported no family history, and 16.1% were unsure. The responses were consistent between groups. Finally, approximately one-quarter of participants (25.1%) identified as being a dementia care partner, with a higher proportion in the intervention group (27.9%) compared to the control group (22.0%)."

16-ii) Primary analysis should be intent-to-treat

Primary analysis should be intent-to-treat, secondary analyses could include comparing only "users", with the appropriate caveats that this is no longer a randomized sample (see 18-i).

	1	2	3	4	5	
subitem not at all important	○	○	○	○	○	essential

Does your paper address subitem 16-ii?

Copy and paste relevant sections from the manuscript (include quotes in quotation marks "like this" to indicate direct quotes from your manuscript), or elaborate on this item by providing additional information not in the ms, or briefly explain why the item is not applicable/relevant for your study

"These models were run for both intention-to-treat (ITT) and per-protocol (PP) group allotment. "

17a) For each primary and secondary outcome, results for each group, and the estimated effect size and its precision (such as 95% confidence interval)

Does your paper address CONSORT subitem 17a? *

Copy and paste relevant sections from the manuscript (include quotes in quotation marks "like this" to indicate direct quotes from your manuscript), or elaborate on this item by providing additional information not in the ms, or briefly explain why the item is not applicable/relevant for your study

"The results of the linear mixed effects model revealed no significant difference between groups in baseline knowledge, intentions to engage in health behaviours, or health behaviours' scores. "

"Figure 4: LMM estimates with 95% CI for knowledge score by: group (panel A), level of education (panel B), and dementia history/care partner (panel C). "

17a-i) Presentation of process outcomes such as metrics of use and intensity of use

In addition to primary/secondary (clinical) outcomes, the presentation of process outcomes such as metrics of use and intensity of use (dose, exposure) and their operational definitions is critical. This does not only refer to metrics of attrition (13-b) (often a binary variable), but also to more continuous exposure metrics such as “average session length”. These must be accompanied by a technical description how a metric like a “session” is defined (e.g., timeout after idle time) [1] (report under item 6a).

	1	2	3	4	5	
subitem not at all important	○	○	○	○	○	essential

Does your paper address subitem 17a-i?

Copy and paste relevant sections from the manuscript (include quotes in quotation marks "like this" to indicate direct quotes from your manuscript), or elaborate on this item by providing additional information not in the ms, or briefly explain why the item is not applicable/relevant for your study

"Metrics related to participant engagement with the e-learning programs are presented in Table 2. Overall, participants had a high e-learning lesson completion rate of 93% for both the intervention and control groups suggesting engagement with the content. Participants spent roughly the expected time on the e-learning lessons: an average of 31 minutes and 52 seconds on the intervention lesson (estimated time to complete was 30-35 minutes), and an average of 26 minutes and 11 seconds on the control lesson (estimated time to complete was 15-20 minutes). The microlearning email campaign was successful in both groups with an open rate of 80.5% for the intervention group and 78.6% for the control group, which is well above MailChimp’s reported industry average open rate of 20.6% for email campaigns. "

17b) For binary outcomes, presentation of both absolute and relative effect sizes is recommended

Does your paper address CONSORT subitem 17b? *

Copy and paste relevant sections from the manuscript (include quotes in quotation marks "like this" to indicate direct quotes from your manuscript), or elaborate on this item by providing additional information not in the ms, or briefly explain why the item is not applicable/relevant for your study

Not applicable, no binary outcomes.

18) Results of any other analyses performed, including subgroup analyses and adjusted analyses, distinguishing pre-specified from exploratory

Does your paper address CONSORT subitem 18? *

Copy and paste relevant sections from the manuscript (include quotes in quotation marks "like this" to indicate direct quotes from your manuscript), or elaborate on this item by providing additional information not in the ms, or briefly explain why the item is not applicable/relevant for your study

"In addition to the predefined variables (group, time, a group*time interaction, age group, education and gender) the final model for knowledge of dementia risk factors included an interaction between education and time and family history of dementia/care partner of someone with dementia with time. Results of this model demonstrated both the intervention and control groups had significant increases in mean knowledge scores relative to baseline at T2 and T3. The change from baseline for the intervention group was statistically significantly greater relative to the control group at both T2 and T3 (Table 3; Figure 4 panel A). "

18-i) Subgroup analysis of comparing only users

A subgroup analysis of comparing only users is not uncommon in ehealth trials, but if done, it must be stressed that this is a self-selected sample and no longer an unbiased sample from a randomized trial (see 16-iii).

	1	2	3	4	5	
subitem not at all important	◯	◯	◯	◯	◯	essential

Does your paper address subitem 18-i?

Copy and paste relevant sections from the manuscript (include quotes in quotation marks "like this" to indicate direct quotes from your manuscript), or elaborate on this item by providing additional information not in the ms, or briefly explain why the item is not applicable/relevant for your study

Your answer

19) All important harms or unintended effects in each group
(for specific guidance see CONSORT for harms)

Does your paper address CONSORT subitem 19? *

Copy and paste relevant sections from the manuscript (include quotes in quotation marks "like this" to indicate direct quotes from your manuscript), or elaborate on this item by providing additional information not in the ms, or briefly explain why the item is not applicable/relevant for your study

There were no harms or unintended effects in either group. We noted that our control group benefited from the e-learning more than initially anticipated.

19-i) Include privacy breaches, technical problems

Include privacy breaches, technical problems. This does not only include physical “harm” to participants, but also incidents such as perceived or real privacy breaches [1], technical problems, and other unexpected/unintended incidents. “Unintended effects” also includes unintended positive effects [2].

	1	2	3	4	5	
subitem not at all important	○	○	○	○	○	essential

Does your paper address subitem 19-i?

Copy and paste relevant sections from the manuscript (include quotes in quotation marks "like this" to indicate direct quotes from your manuscript), or elaborate on this item by providing additional information not in the ms, or briefly explain why the item is not applicable/relevant for your study

There were no privacy breaches or technical problems.

19-ii) Include qualitative feedback from participants or observations from staff/researchers

Include qualitative feedback from participants or observations from staff/researchers, if available, on strengths and shortcomings of the application, especially if they point to unintended/unexpected effects or uses. This includes (if available) reasons for why people did or did not use the application as intended by the developers.

	1	2	3	4	5	
subitem not at all important	○	○	○	○	○	essential

Does your paper address subitem 19-ii?

Copy and paste relevant sections from the manuscript (include quotes in quotation marks "like this" to indicate direct quotes from your manuscript), or elaborate on this item by providing additional information not in the ms, or briefly explain why the item is not applicable/relevant for your study

Qualitative quotes were included in the manuscript.

DISCUSSION

22) Interpretation consistent with results, balancing benefits and harms, and considering other relevant evidence

NPT: In addition, take into account the choice of the comparator, lack of or partial blinding, and unequal expertise of care providers or centers in each group

22-i) Restate study questions and summarize the answers suggested by the data, starting with primary outcomes and process outcomes (use)

Restate study questions and summarize the answers suggested by the data, starting with primary outcomes and process outcomes (use).

	1	2	3	4	5	
subitem not at all important	○	○	○	○	○	essential

Does your paper address subitem 22-i? *

Copy and paste relevant sections from the manuscript (include quotes in quotation marks "like this" to indicate direct quotes from your manuscript), or elaborate on this item by providing additional information not in the ms, or briefly explain why the item is not applicable/relevant for your study

"The findings from this randomized controlled trial demonstrate the efficacy of the DementiaRisk internet-based education program in enhancing participants' knowledge of dementia risk and protective factors. These results were statistically significant at both the 4-week and 2-months post-intervention follow-up assessments, reflecting sustained improvements post-intervention."

22-ii) Highlight unanswered new questions, suggest future research

Highlight unanswered new questions, suggest future research.

	1	2	3	4	5	
subitem not at all important	○	○	○	○	○	essential

Does your paper address subitem 22-ii?

Copy and paste relevant sections from the manuscript (include quotes in quotation marks "like this" to indicate direct quotes from your manuscript), or elaborate on this item by providing additional information not in the ms, or briefly explain why the item is not applicable/relevant for your study

"While our results suggest that the intervention increased participants' motivation to change their behaviours, future research should explore the development and validation of standardized measures for assessing dementia-related health behaviour intentions. Furthermore, it is important to acknowledge the 'intention-behaviour gap', where individuals express a willingness to change but do not always follow through with action (49,50). Future studies should consider incorporating follow-up assessments at longer intervals to determine whether increased intentions translate into sustained behaviour change."

20) Trial limitations, addressing sources of potential bias, imprecision, and, if relevant, multiplicity of analyses

20-i) Typical limitations in ehealth trials

Typical limitations in ehealth trials: Participants in ehealth trials are rarely blinded. Ehealth trials often look at a multiplicity of outcomes, increasing risk for a Type I error. Discuss biases due to non-use of the intervention/usability issues, biases through informed consent procedures, unexpected events.

	1	2	3	4	5	
subitem not at all important	○	○	○	○	○	essential

Does your paper address subitem 20-i? *

Copy and paste relevant sections from the manuscript (include quotes in quotation marks "like this" to indicate direct quotes from your manuscript), or elaborate on this item by providing additional information not in the ms, or briefly explain why the item is not applicable/relevant for your study

"To the best of our ability, efforts were made to blind participants to the study hypothesis and allocation group by referring to the study in broad terms, such as 'e-learning about cognitive impairment and promoting brain health' (rather than explicitly 'dementia prevention', for example). The principal investigator [AJL] and data analysts [DH and AS] remained blinded to group allocation throughout the data collection and analysis phases."

21) Generalisability (external validity, applicability) of the trial findings

NPT: External validity of the trial findings according to the intervention, comparators, patients, and care providers or centers involved in the trial

21-i) Generalizability to other populations

Generalizability to other populations: In particular, discuss generalizability to a general Internet population, outside of a RCT setting, and general patient population, including applicability of the study results for other organizations

	1	2	3	4	5	
subitem not at all important	○	○	○	○	○	essential

Does your paper address subitem 21-i?

Copy and paste relevant sections from the manuscript (include quotes in quotation marks "like this" to indicate direct quotes from your manuscript), or elaborate on this item by providing additional information not in the ms, or briefly explain why the item is not applicable/relevant for your study

"Future studies should focus on testing the intervention in more diverse populations, particularly those who are socially disadvantaged or less familiar with online educational tools. Expanding the program to other languages and tailoring content to specific cultural contexts may also enhance its applicability across different demographics.

Additionally, further research could explore the longer-term impact of the program on health behaviours or actual dementia incidence. While the current study demonstrated changes in knowledge, intentions, and short-term behaviours, a longitudinal study would be necessary to determine if these changes can be sustained or translate into a reduced risk of dementia over time."

21-ii) Discuss if there were elements in the RCT that would be different in a routine application setting

Discuss if there were elements in the RCT that would be different in a routine application setting (e.g., prompts/reminders, more human involvement, training sessions or other co-interventions) and what impact the omission of these elements could have on use, adoption, or outcomes if the intervention is applied outside of a RCT setting.

	1	2	3	4	5	
subitem not at all important	○	○	○	○	○	essential

Does your paper address subitem 21-ii?

Copy and paste relevant sections from the manuscript (include quotes in quotation marks "like this" to indicate direct quotes from your manuscript), or elaborate on this item by providing additional information not in the ms, or briefly explain why the item is not applicable/relevant for your study

"The asynchronous nature of the program (any time, any place, self-paced), along with its micro-learning components, provided flexibility for participants to engage with the material at their own pace and convenience. This likely improved completion rates and helped maintain participant engagement throughout the study, as evidenced by the high lesson completion rate for both e-learning programs where 93% of participants who started the lesson viewed it to completion in a reasonable and expected amount of time (an average of 31 minutes, 52 seconds for the intervention lesson and 26 minutes, 11 seconds for the control lesson)."

OTHER INFORMATION

23) Registration number and name of trial registry

Does your paper address CONSORT subitem 23? *

Copy and paste relevant sections from the manuscript (include quotes in quotation marks "like this" to indicate direct quotes from your manuscript), or elaborate on this item by providing additional information not in the ms, or briefly explain why the item is not applicable/relevant for your study

ClinicalTrials.gov NCT05383118; May 18, 2022.

24) Where the full trial protocol can be accessed, if available

Does your paper address CONSORT subitem 24? *

Cite a Multimedia Appendix, other reference, or copy and paste relevant sections from the manuscript (include quotes in quotation marks "like this" to indicate direct quotes from your manuscript), or elaborate on this item by providing additional information not in the ms, or briefly explain why the item is not applicable/relevant for your study

https://www.researchprotocols.org/2025/1/e64718 - also cited within the paper.

25) Sources of funding and other support (such as supply of drugs), role of funders

Does your paper address CONSORT subitem 25? *

Copy and paste relevant sections from the manuscript (include quotes in quotation marks "like this" to indicate direct quotes from your manuscript), or elaborate on this item by providing additional information not in the ms, or briefly explain why the item is not applicable/relevant for your study

"This work was supported by the research team at the Division of e-Learning Innovation at McMaster University. Funding was provided by the Public Health Agency of Canada Dementia Strategic Fund. AJL receives support through the John R Evans Chair in Health Sciences Educational Research and Instructional Development, Faculty of Health Sciences, McMaster University. "

X27) Conflicts of Interest (not a CONSORT item)

X27-i) State the relation of the study team towards the system being evaluated

In addition to the usual declaration of interests (financial or otherwise), also state the relation of the study team towards the system being evaluated, i.e., state if the authors/evaluators are distinct from or identical with the developers/sponsors of the intervention.

	1	2	3	4	5	
subitem not at all important	○	○	○	○	○	essential

Does your paper address subitem X27-i?

Copy and paste relevant sections from the manuscript (include quotes in quotation marks "like this" to indicate direct quotes from your manuscript), or elaborate on this item by providing additional information not in the ms, or briefly explain why the item is not applicable/relevant for your study

"This work was supported by the research team at the Division of e-Learning Innovation at McMaster University. Funding was provided by the Public Health Agency of Canada Dementia Strategic Fund. AJL receives support through the John R Evans Chair in Health Sciences Educational Research and Instructional Development, Faculty of Health Sciences, McMaster University. "

"AJL, RS, and McMaster University are co-owners of DementiaRisk."

About the CONSORT EHEALTH checklist

As a result of using this checklist, did you make changes in your manuscript? *

- ○ yes, major changes
- ○ yes, minor changes
- ◉ no

What were the most important changes you made as a result of using this checklist?

Your answer

How much time did you spend on going through the checklist INCLUDING making changes in your manuscript *

It took approximately 3 hours to go through this checklist and find the appropriate parts of the manuscript to cite.

As a result of using this checklist, do you think your manuscript has improved? *

- (•) yes
- ( ) no
- ( ) Other:

Would you like to become involved in the CONSORT EHEALTH group?

This would involve for example becoming involved in participating in a workshop and writing an "Explanation and Elaboration" document

- ( ) yes
- (•) no
- ( ) Other:

Clear selection

Any other comments or questions on CONSORT EHEALTH

Your answer

STOP - Save this form as PDF before you click submit

To generate a record that you filled in this form, we recommend to generate a PDF of this page (on a Mac, simply select "print" and then select "print as PDF") before you submit it.

When you submit your (revised) paper to JMIR, please upload the PDF as supplementary file.

Don't worry if some text in the textboxes is cut off, as we still have the complete information in our database. Thank you!

Final step: Click submit !

Click submit so we have your answers in our database!

Submit

Clear form

Never submit passwords through Google Forms.

This form was created outside of your domain. - Contact form owner - Terms of Service - Privacy Policy

Does this form look suspicious? Report

Google Forms
